# Supplementary material for: Longitudinal single cell atlas identifies complex temporal relationship between type I interferon response and COVID-19 severity
Source: Nat Commun. 2024 Jan 18;15:567. doi: 10.1038/s41467-023-44524-0 (PMC10796319; doi:10.1038/s41467-023-44524-0)
Supplement: Supplementary file 1 — Supplementary Information [file 41467_2023_44524_MOESM1_ESM.pdf]

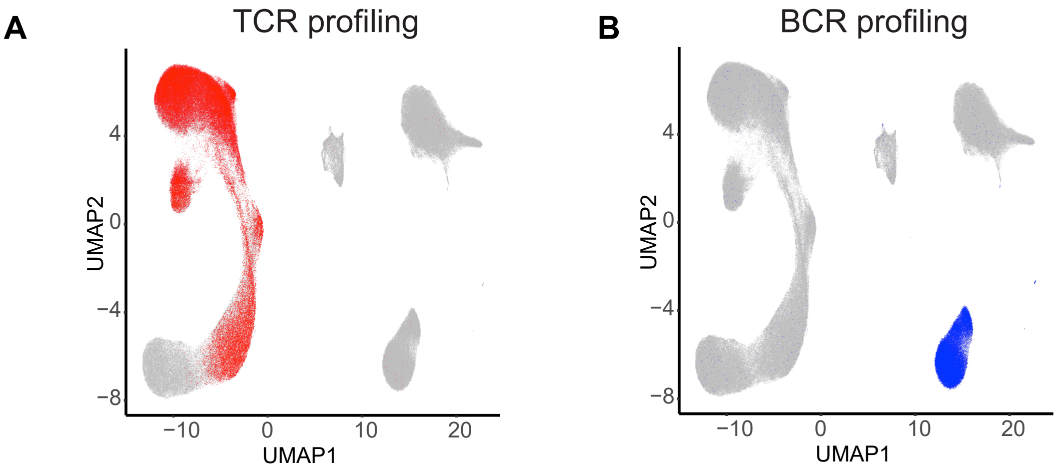


**Supplementary Figure 1. TCR and BCR profiling.** UMAP plots show the detected TCRs (red in **A**) and BCRs (blue in **B**).

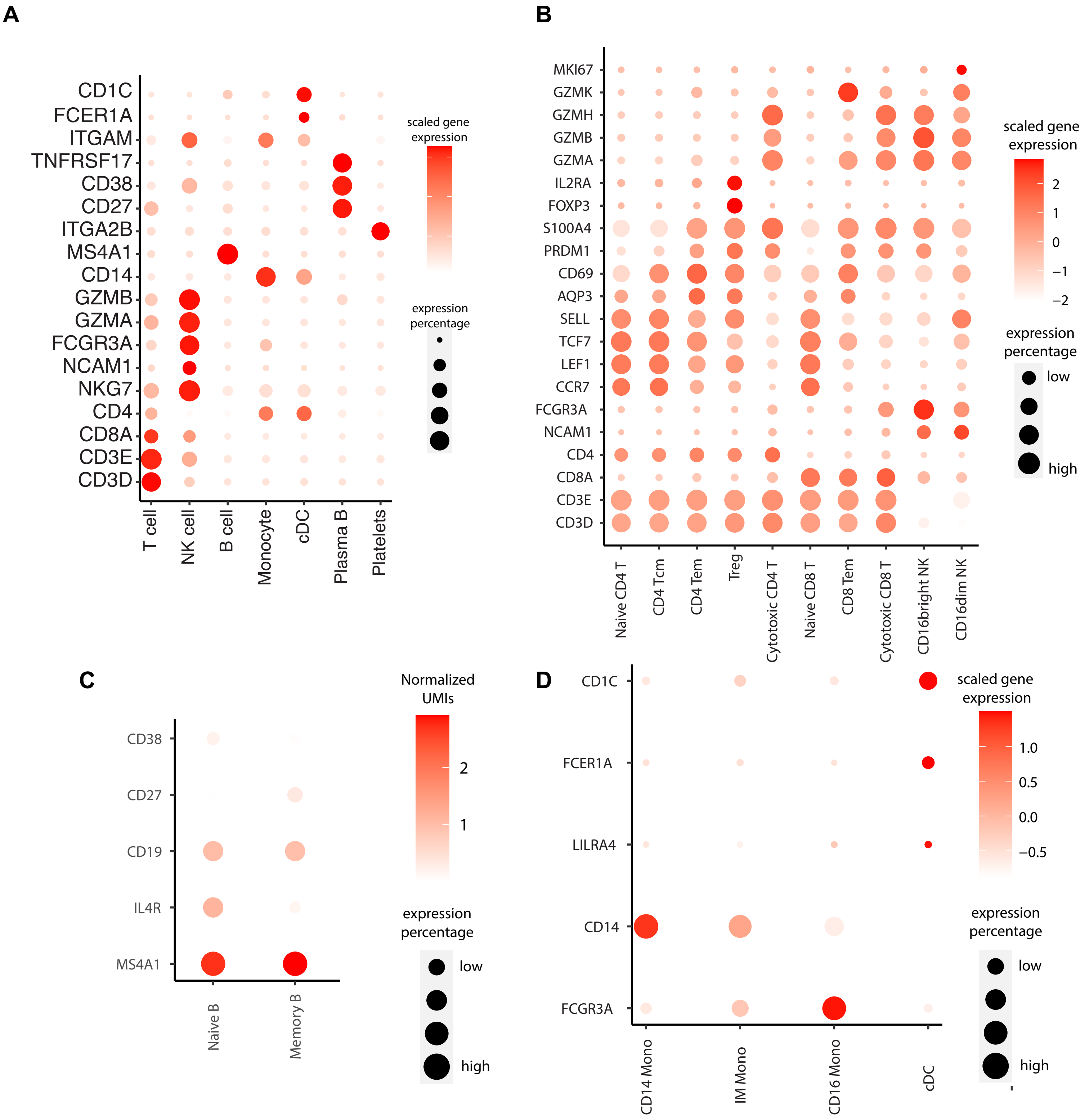


**Supplementary Figure 2. Marker gene profiling across major cell types.** Bubble plots show expression levels of marker genes across cell types: all major cell types (**A**), T, NK cells (**B**), B cells (**C**) and myeloid cells (**D**). The bubble size indicates gene expression percentage, while color intensity represents gene expression level.

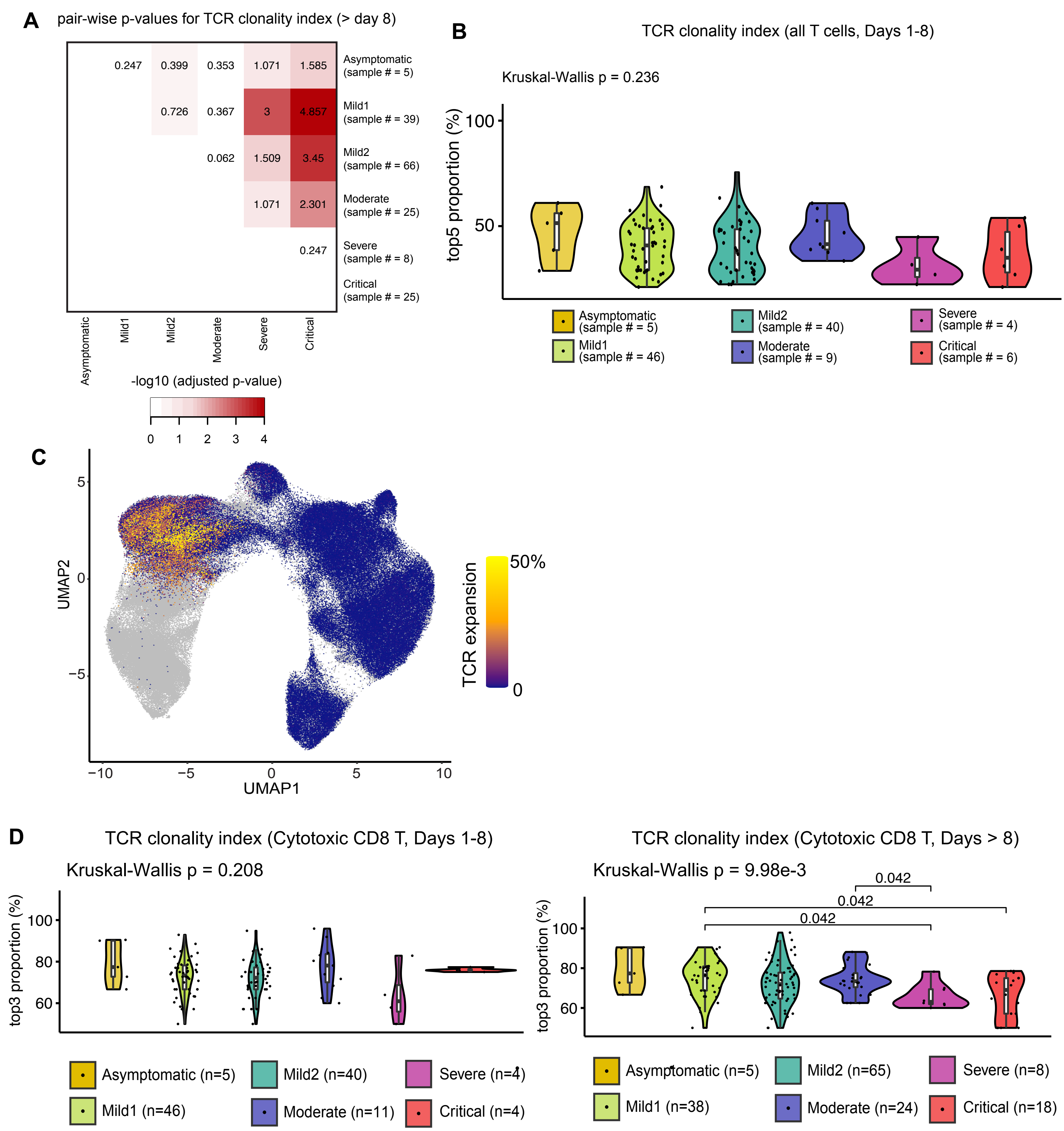


**Supplementary Figure 3. TCR clonality index across clinical severities. A**) The heatmap shows the Benjamini-Hochberg adjusted *p*-values using the Wilcoxon rank-sum test between samples in two severities (> day 8 after symptom onset). **B**) T cell clonality index (fraction of T cells derived from the 5 most abundant TCR clones in top 50 TCRs); Days 1-8), estimated using the single cell immune profiling assay. p-value: Kruskal–Wallis test. **C)** The UMAP shows the T, NK cells colored by TCR clonality proportion. If TCR is not detected, cell is colored as grey. If TCR is detected, cell is colored by TCR clonality proportion in its corresponding sample. For example, if the proportion value for a cell is 50%, it means that its TCR is observed 50% of all detected TCRs in the corresponding sample. **D)** Violin plots show the percentages of top 3 most abundant TCRs in top 6 TCRs across different severities for Cytotoxic CD8 T (samples collected from days 1-8 on the left end and > days 8 on the right side). *P*-value (Benjamini-Hochberg adjusted) on the top of each bar (only p-value < 0.05 shown): Wilcoxon rank-sum test; *P*-value in the upper left corner: Kruskal-Wallis test. Box plots show the median (centre line), 25th and 75th percentile (lower and upper boundary), with 1.5x inter quartile range indicated by whiskers and outliers shown as individual data points.

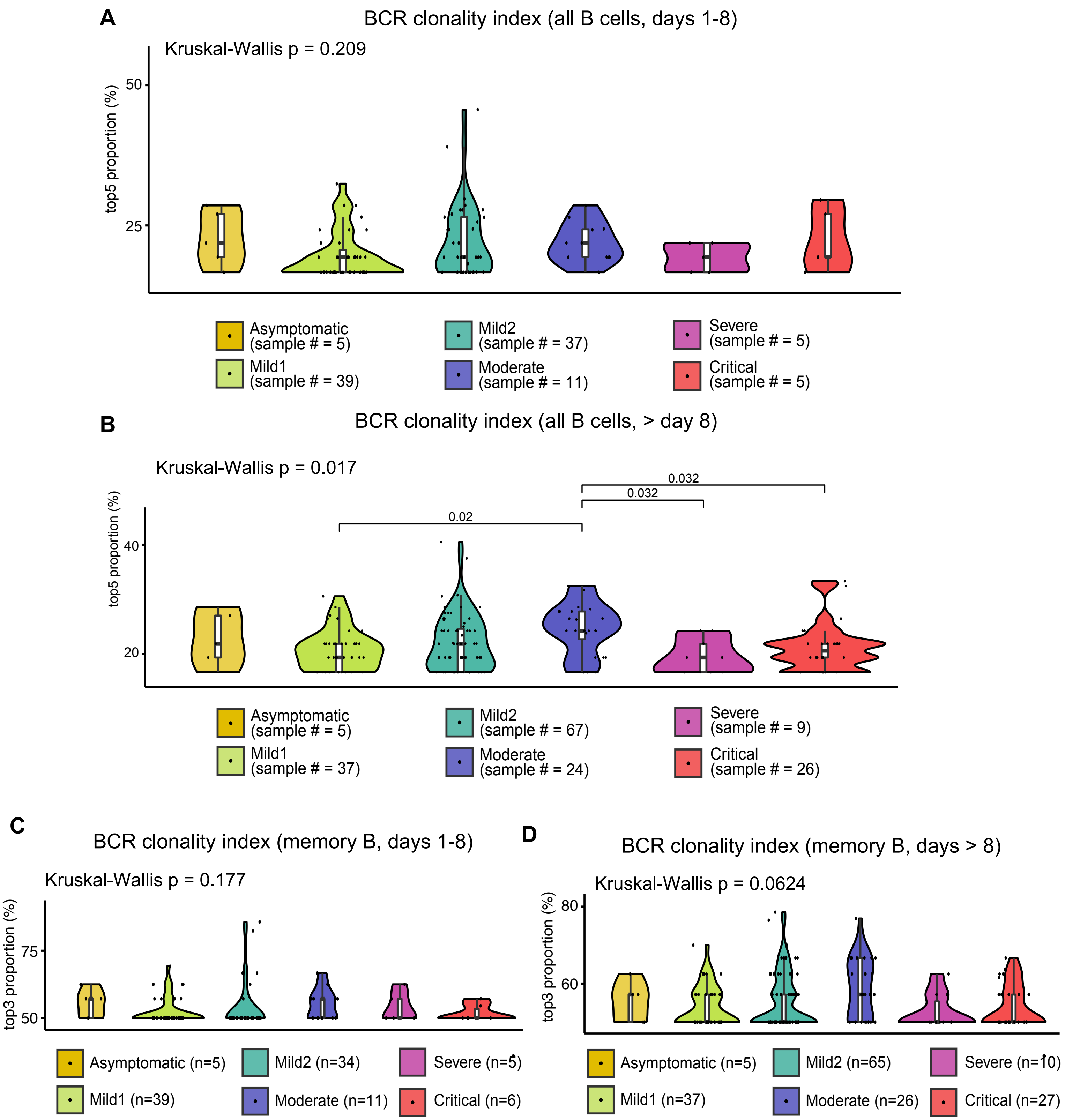


**Supplementary Figure 4. BCR clonality index across clinical severities. A-B)** Violin plots show the percentages of top 5 most abundant BCRs in top 30 BCRs across different severities for all B cells, samples collected in days 1-8 **(A)** and > day 8 **(B)** after symptom onset. *P*-value (Benjamini-Hochberg adjusted) on the top of each bar (only p-value < 0.05 shown): Wilcoxon rank-sum test; *P*-value in the upper left corner: Kruskal-Wallis test. **C-D)** Violin plots show the percentages of top 3 most abundant BCRs in top 6 BCRs across different severities for memory B cells, samples collected in days 1-8 **(C)** and > day 8 **(D)** after symptom onset. *P*-value (Benjamini-Hochberg adjusted) on the top of each bar (only p-value < 0.05 shown): Wilcoxon rank-sum test; *P*-value in the upper left corner: Kruskal-Wallis test. Box plots show the median (centre line), 25th and 75th percentile (lower and upper boundary), with 1.5x inter quartile range indicated by whiskers and outliers shown as individual data points.

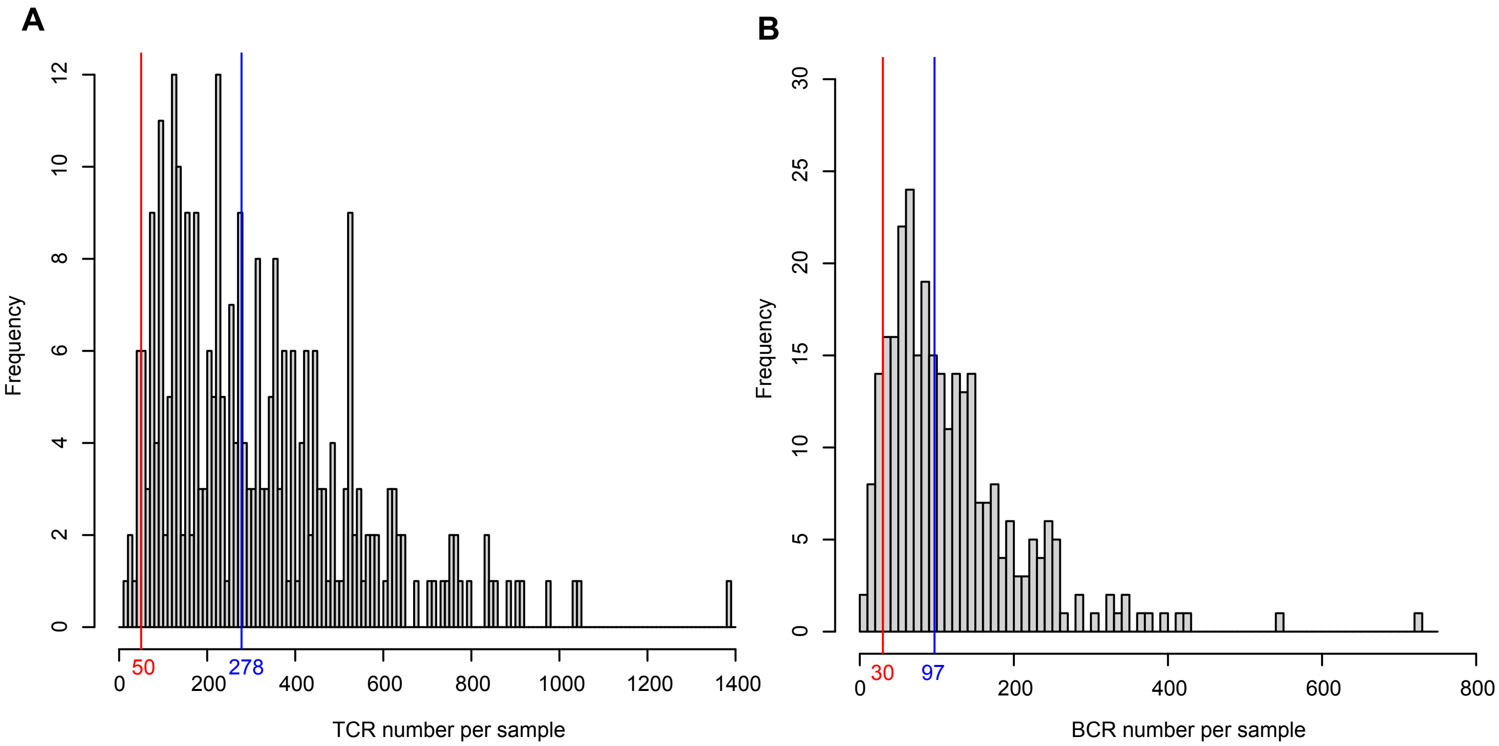


**Supplementary Figure 5. TCR and BCR numbers per sample.** Bar plots show the frequency distributions of detected TCRs **(A)** and BCRs **(B)** across samples. Red lines denote the mininum numbers of detected TCRs/BCRs required for a sample to be used in our downstream anlaysis, while blue lines indicate the median numbers of detected TCRs/BCRs across all samples.

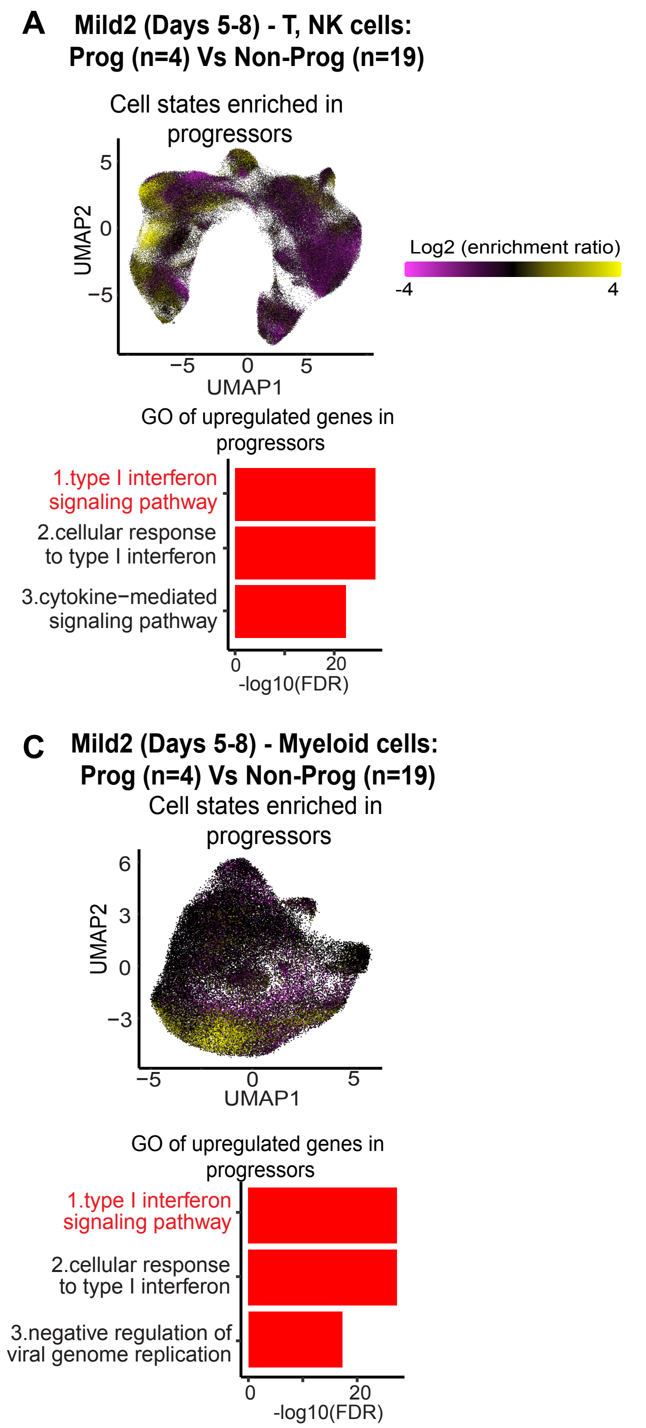


**Supplementary Figure 6.** UMAP representations show the cell-state enrichment fold change between Mild2 Progressors and Non-Progressors in T, NK cells (A), B cells (B), and myeloid cells (C). Yellow: cell states (gene expression neighborhoods) enriched in Progressors. Magenta: cell states depleted in Progressors. Red bar plots: enriched Gene Ontology (GO) terms associated with the union across all cell subtypes of genes up-regulated in Progressors.

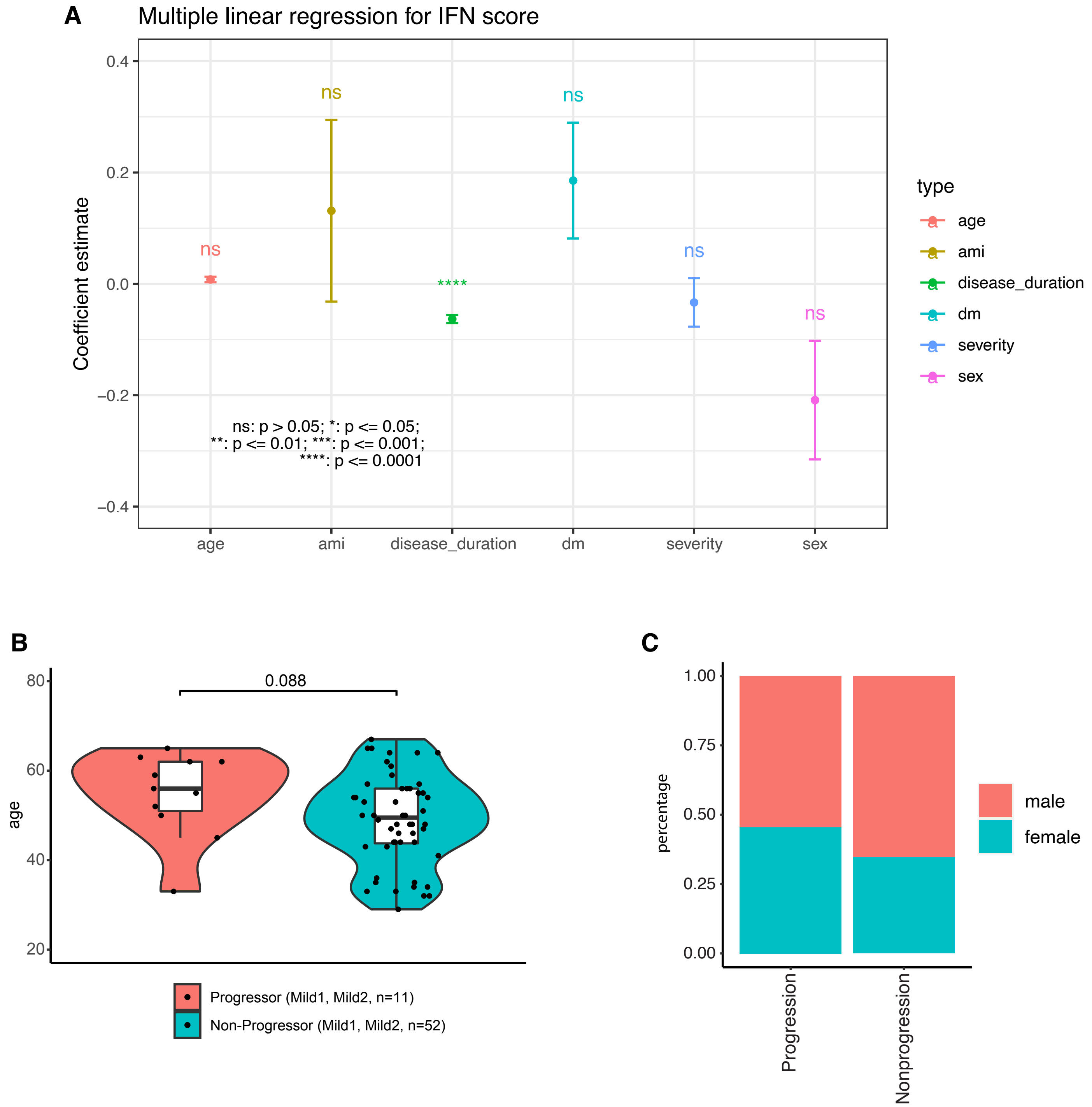


**Supplementary Figure 7. A)** Regression coefficients and standard errors from a multivariate linear regression model for predicting the prognostic ISG expression score based on 6 variables: age, sex, disease duration, disease severity, ami and dm. **B)** Violin plot shows the comparison of age between Progressors and Non-Progressors. The value above bar between two violins is the p-value calculated using Student's t test. Box plots show the median (centre line), 25th and 75th percentile (lower and upper boundary), with 1.5x inter quartile range indicated by whiskers and outliers shown as individual data points. **C)** Barplot shows the percentages of male and female individuals in Progressors and Non-Progressors.

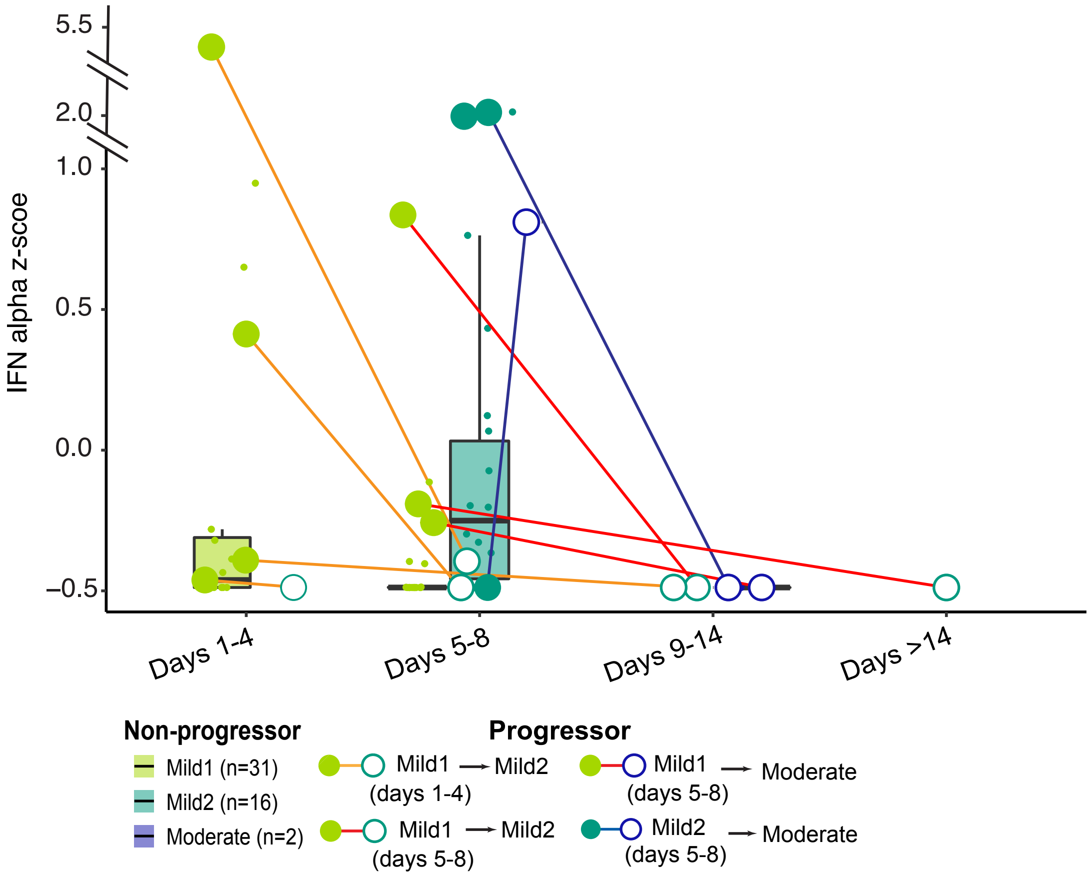


**Supplementary Figure 8.** Plasma IFN-alpha protein expression z-score of Mild Progressor (circles) and Non-Progressor (box plots) samples, grouped by disease severity and duration. Filled circles: baseline samples of Progressors. Empty circles: second samples of Progressors. Box plots show the median (centre line), 25th and 75th percentile (lower and upper boundary), with 1.5x inter quartile range indicated by whiskers and outliers shown as individual data points.

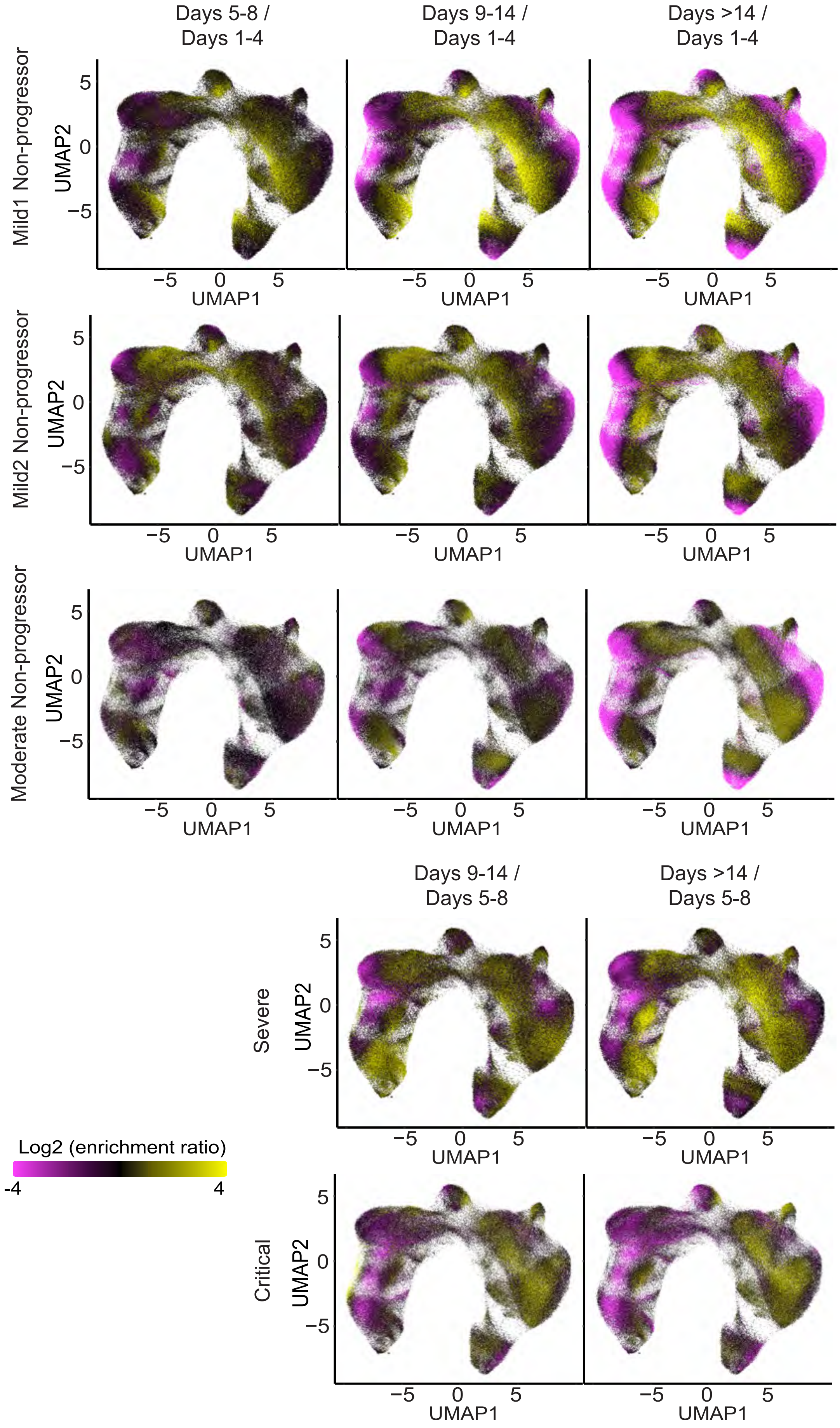


**Supplementary Figure 9. Temporal dynamics of T and NK cell states in Non-Progressors.** The UMAP plots show the cell state enrichment fold changes between each temporal stage and the first reference state for all severities. For Mild1, Mild2 and Moderate,samples from days 1-4 were used as reference, while for Severe and Critical, samples from days 5-8 were used as reference (only 1 sample in days 1-4). Yellow color denotes cell states enriched in the specific temporal stage compared with the first stage, while magenta indicates cell states depleted in the corresponding temporal stage.

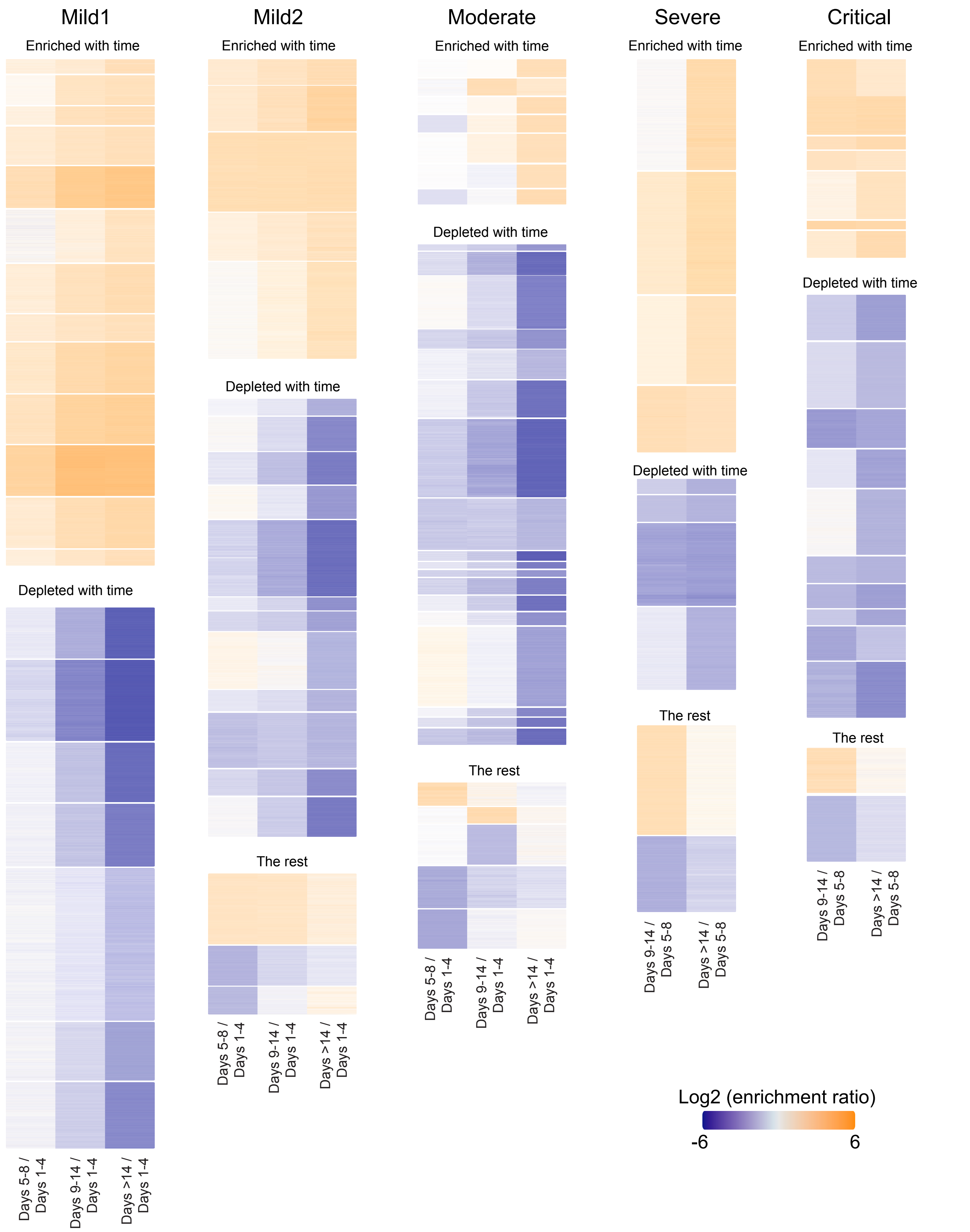


**Supplementary Figure 10. Clusters of T and NK cell state enrichments in Non-Progressors.** Heatmaps show the clusters of cell state enrichment fold changes between each temporal stage and the first reference stage for all disease severities. If the Pearson correlation for a cluster is greater than 0.5, it is considered enriched with time. If the Pearson correlation for a cluster is less than -0.5, it's considered depleted with time. If the Pearson correlation for a cluster is between -0.5 and 0.5, it is grouped under the rest.

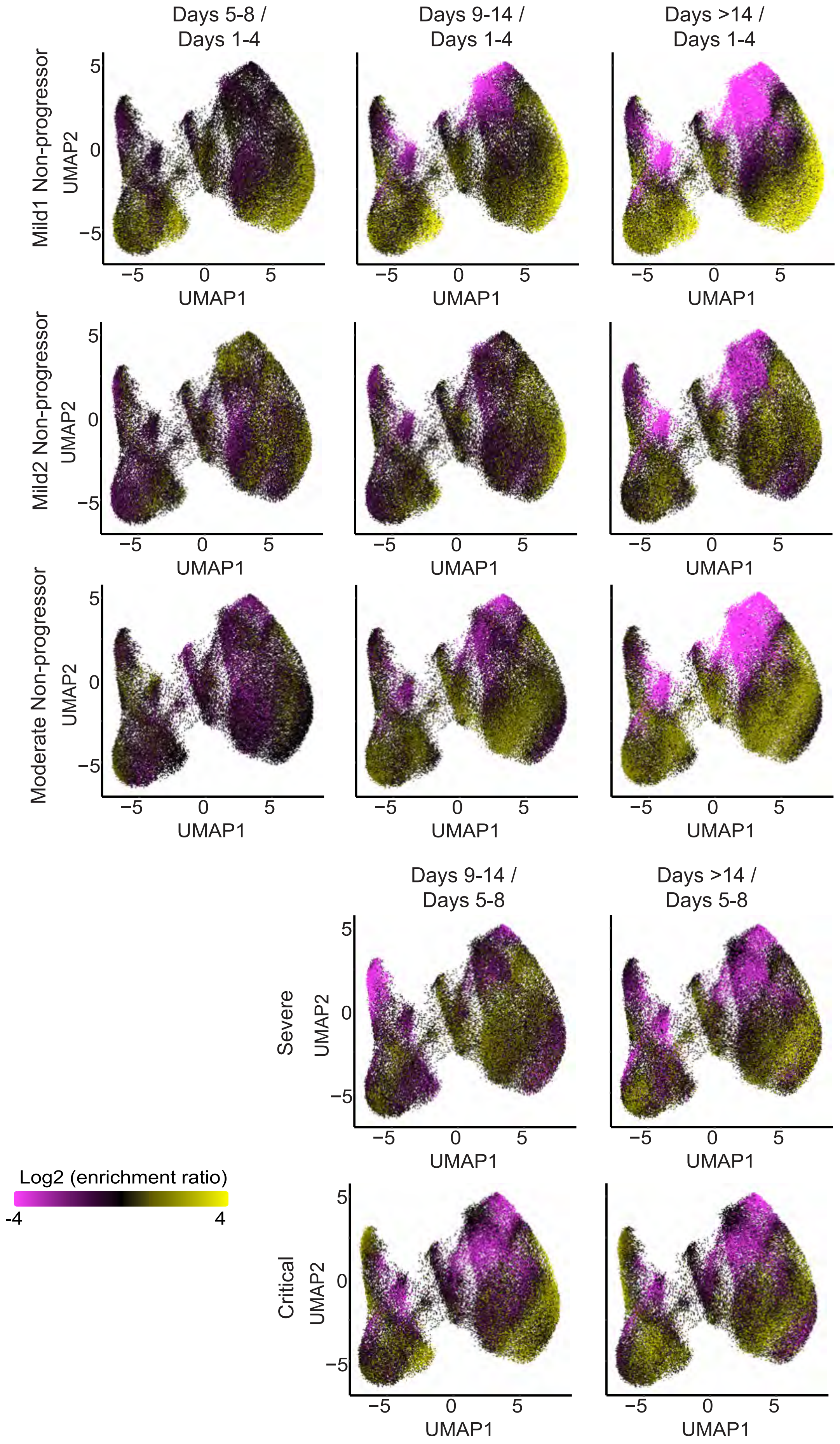


**Supplementary Figure 11. Temporal dynamics of B cell states in Non-Progressors.** The UMAP plots show the cell state enrichment fold changes between each temporal stage and the first reference state for all severities. For Mild1, Mild2 and Moderate, samples from days 1-4 were used as reference, while for Severe and Critical, samples from days 5-8 were used as reference (only 1 sample in days 1-4). Yellow color denotes cell states enriched in the specific temporal stage compared with the first stage, while magenta indicates cell states depleted in the corresponding temporal stage.

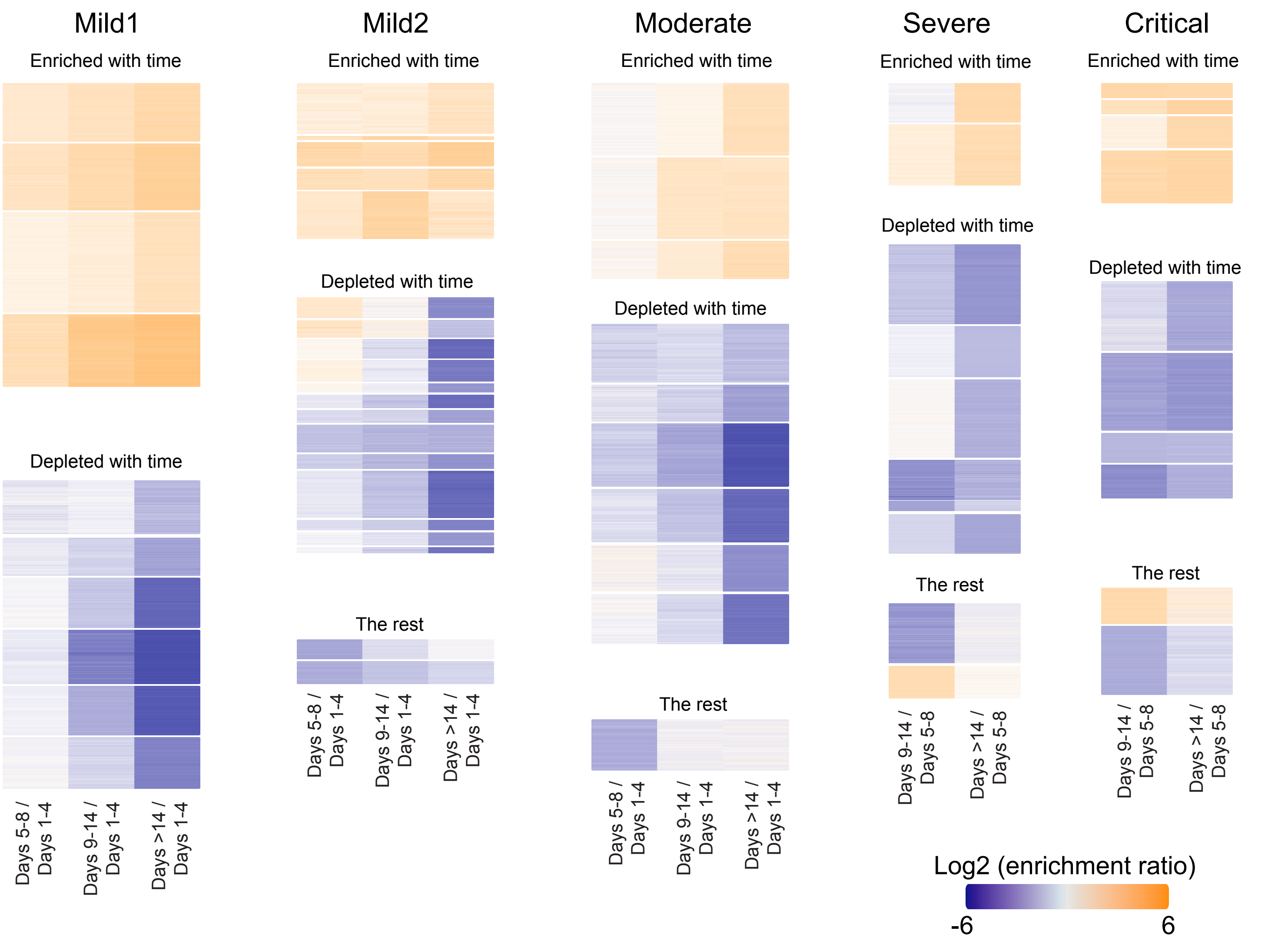


**Supplementary Figure 12. Clusters of B cell state enrichments in Non-Progressors.** Heatmaps show the clusters of cell state enrichment fold changes between each temporal stage and the first reference stage for all disease severities. If the Pearson correlation for a cluster is greater than 0.5, it is considered enriched with time. If the Pearson correlation for a cluster is less than -0.5, it's considered depleted with time. If the Pearson correlation for a cluster is between -0.5 and 0.5, it is grouped under the rest.

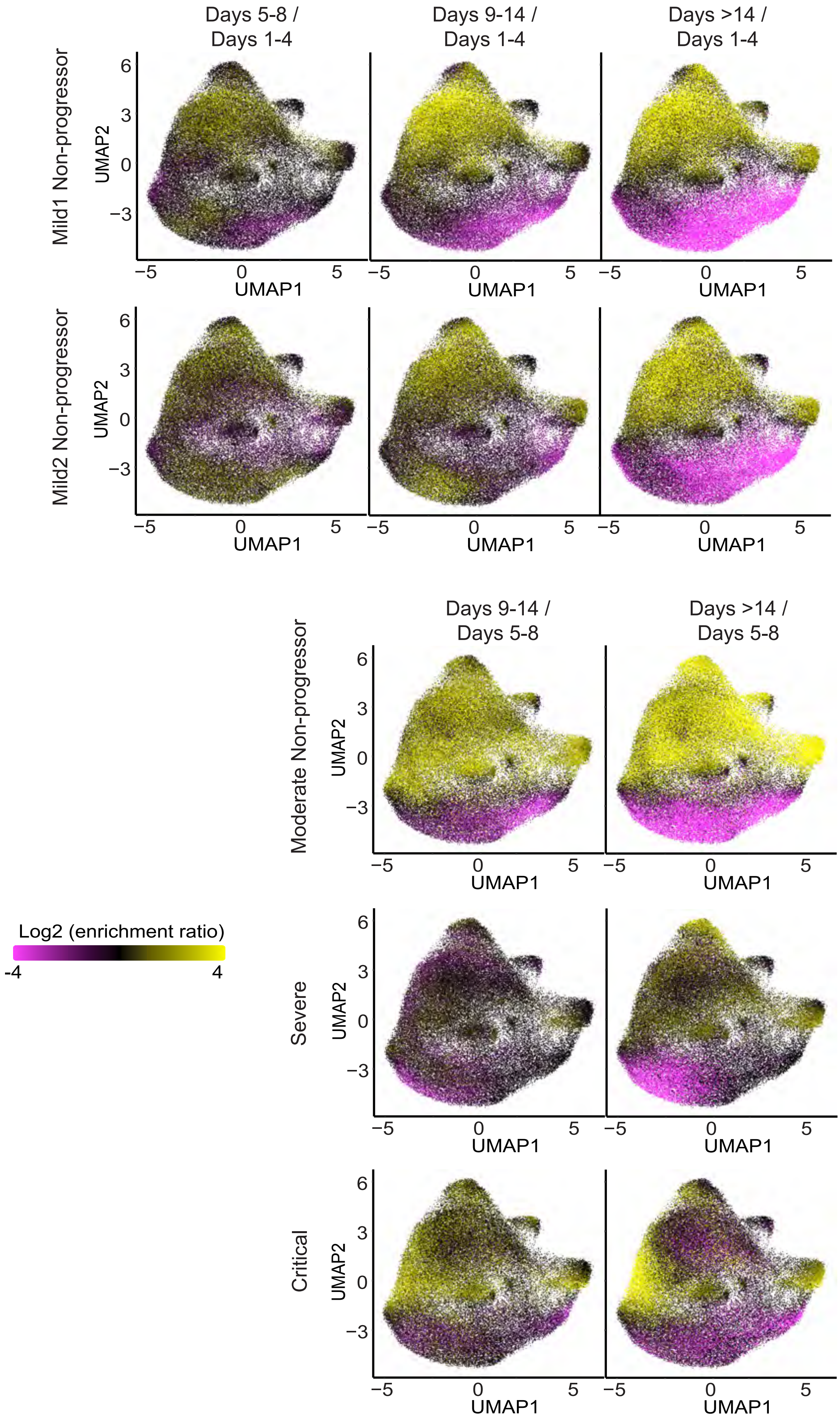


**Supplementary Figure 13. Temporal dynamics of myeloid cell states in Non-Progressors.** The UMAP plots show the cell state enrichment fold changes between each temporal stage and the first reference stage for all severities. For Mild1 and Mild2, samples from days 1-4 were used as reference, while for Severe and Critical, samples from days 5-8 were used as reference (only 1 sample in days 1-4). Even though there were 2 Moderate samples in days 1-4, only 92 cells were in that temporal stage. Thus samples from days 5-8 were used as reference for Moderate. Yellow color denotes cell states enriched in the corresponding temporal stage compared with the first state, while magenta indicates cell states depleted in the corresponding temporal stage.

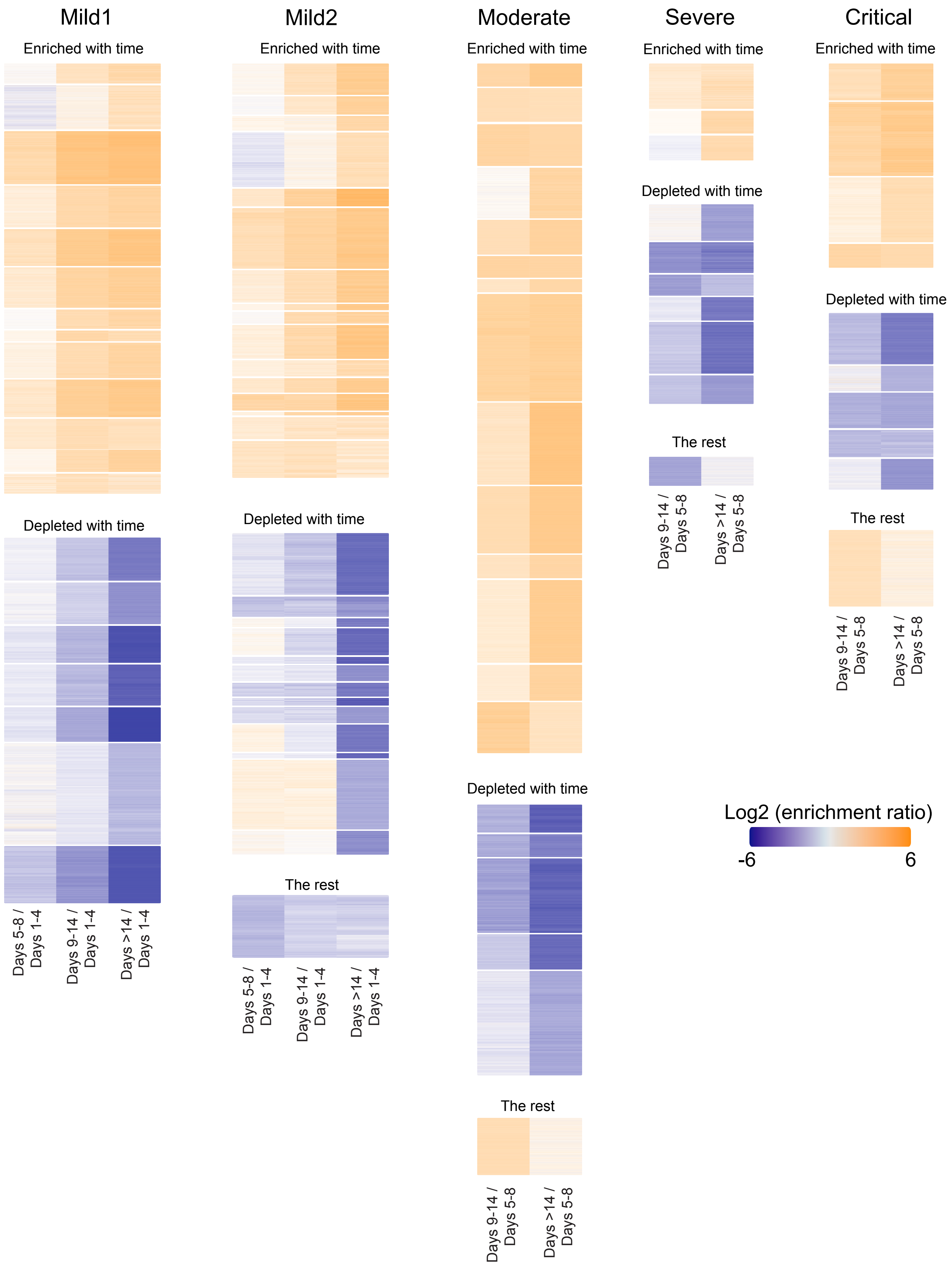


**Supplementary Figure 14. Clusters of myeloid cell state enrichments in Non-Progressors.** Heatmaps show the clusters of cell state enrichment fold changes between each temporal stage and the first reference stage for all disease severities. If the Pearson correlation for a cluster is greater than 0.5, it is considered enriched with time. If the Pearson correlation for a cluster is less than -0.5, it's considered depleted with time. If the Pearson correlation for a cluster is between -0.5 and 0.5, it is grouped under the rest.

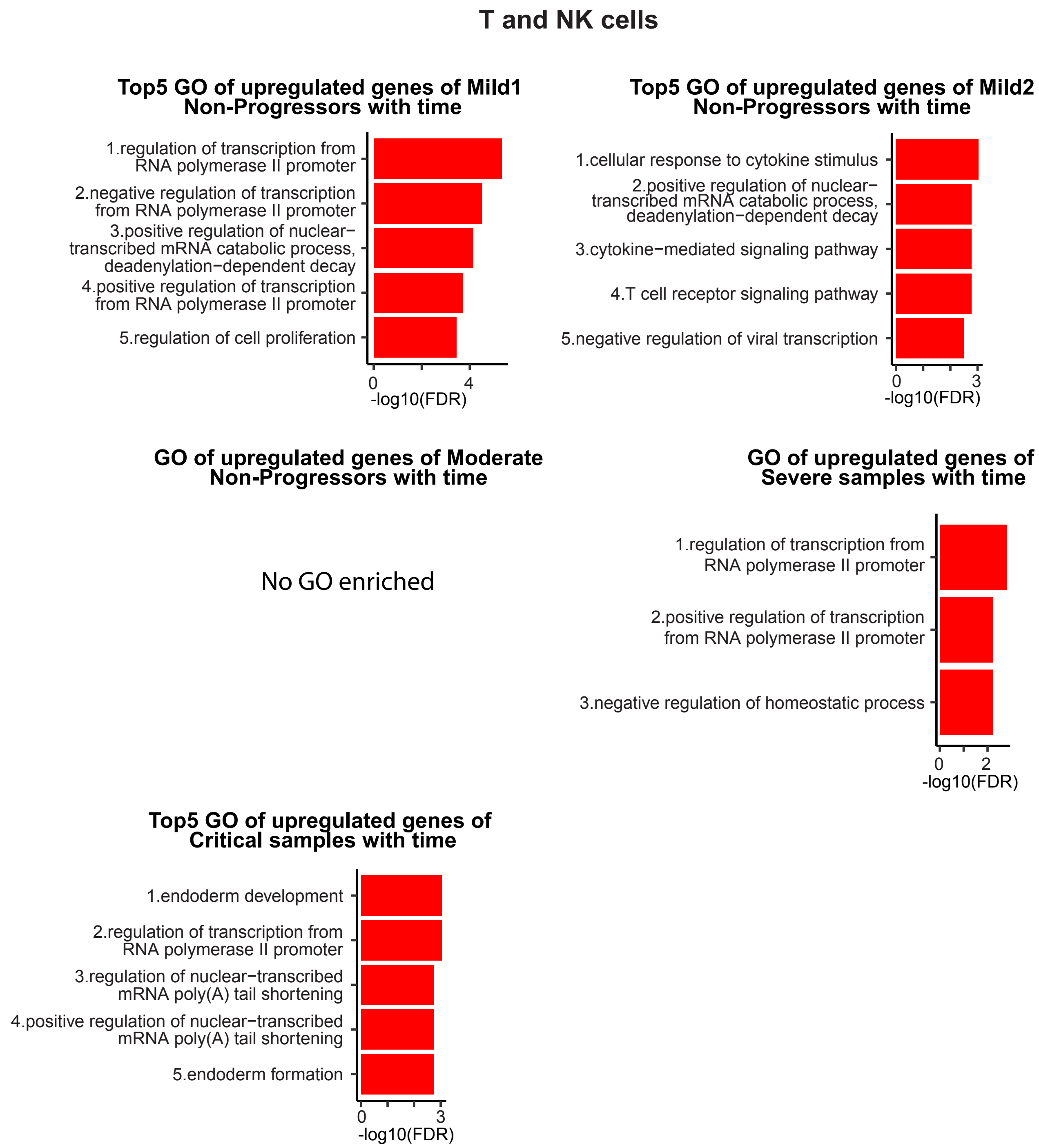


**Supplementary Figure 15. GO enrichment analysis of upregulated genes in T and NK cells of Non-Progressors with time.** Bar plots show the top GO terms associated with the upregulated genes in Non-Progressors with time at all disease severities.

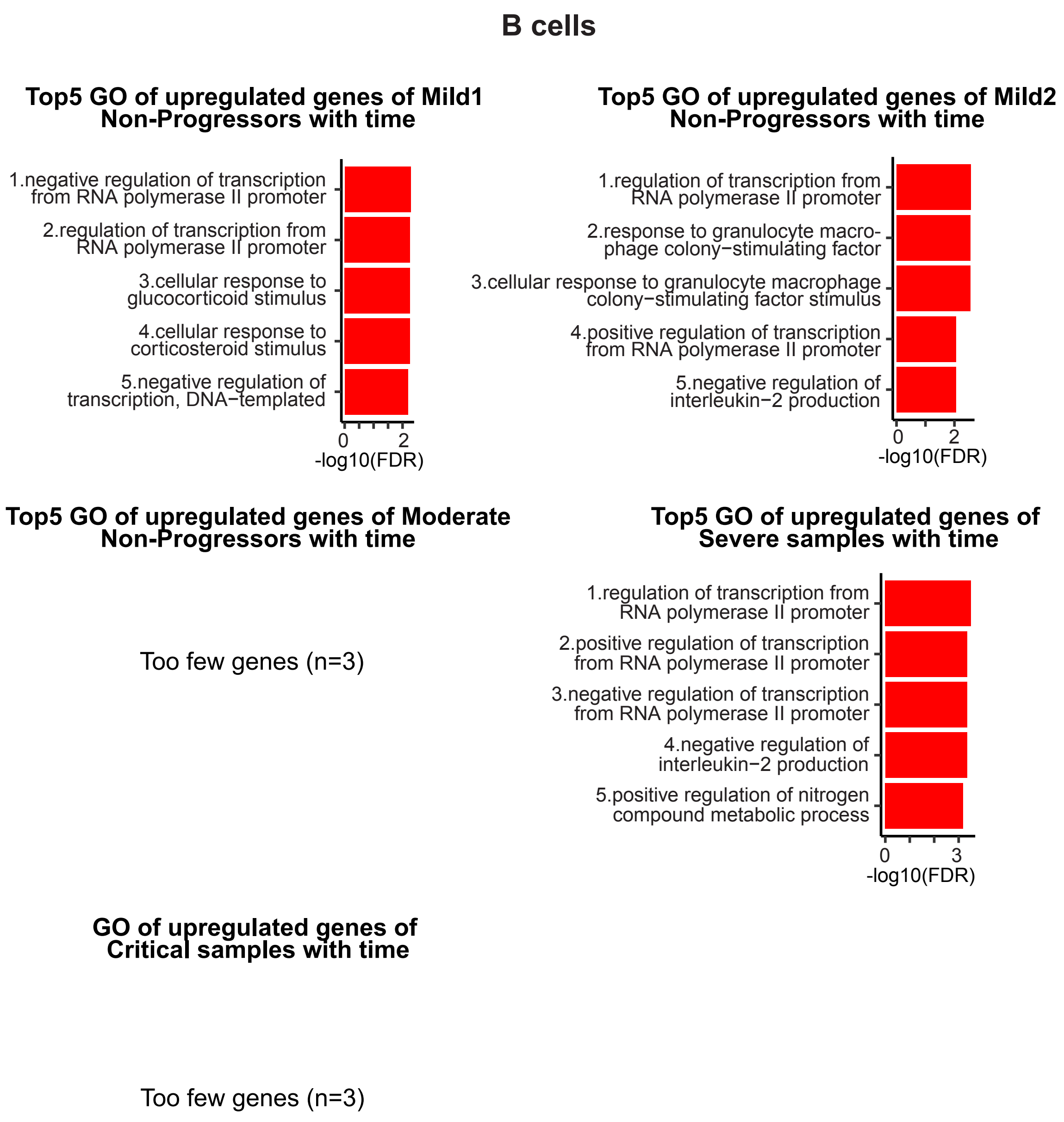


**Supplementary Figure 16. GO enrichment analysis of upregulated genes in B cells of Non-Progressors with time.** Bar plots show the top GO terms associated with the upregulated genes in Non-Progressors with time at all disease severities.

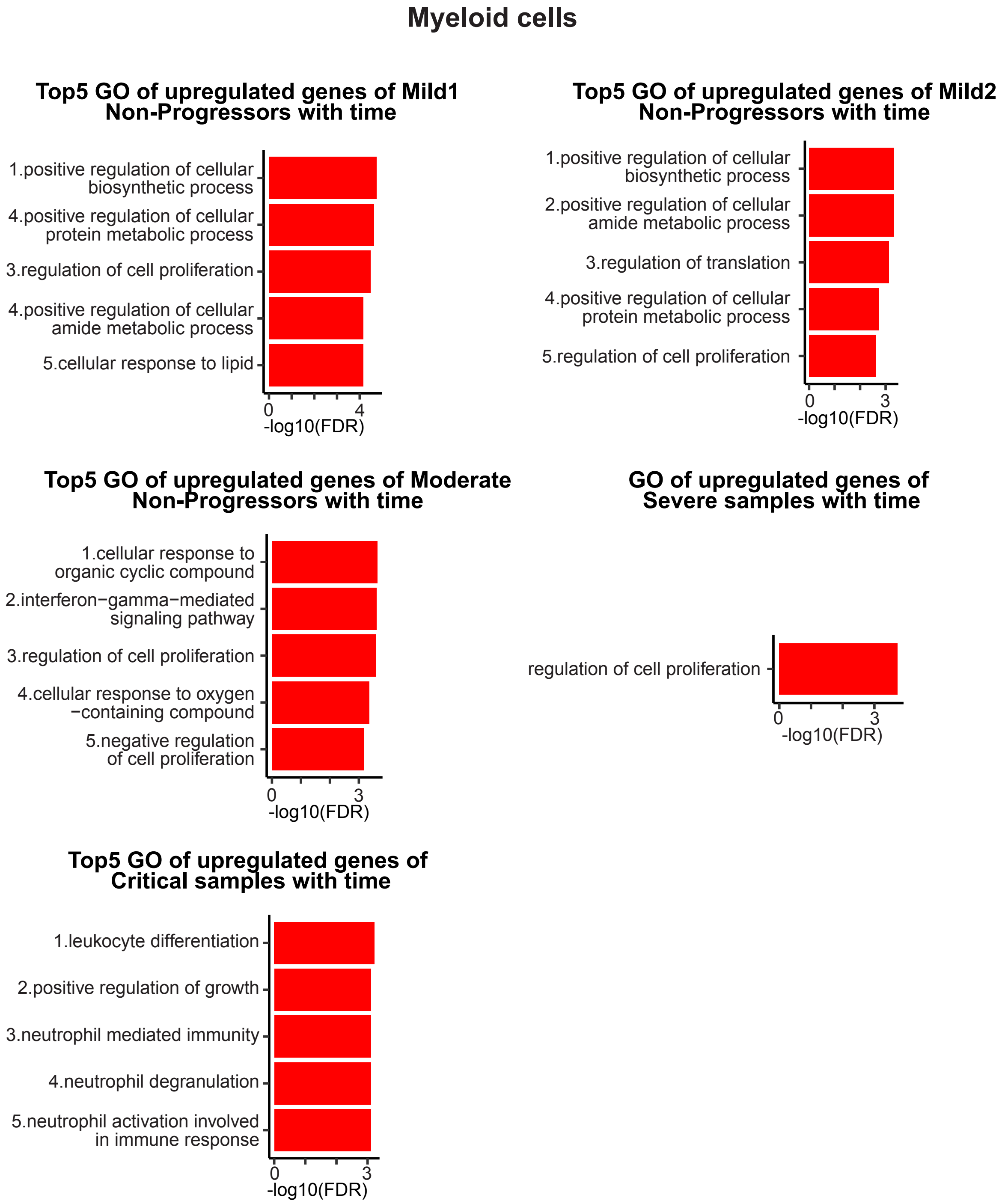


**Supplementary Figure 17. GO enrichment analysis of upregulated genes in myeloid cells of Non-Progressors with time.** Bar plots show the top GO terms associated with the upregulated genes in Non-Progressors with time at all disease severities.

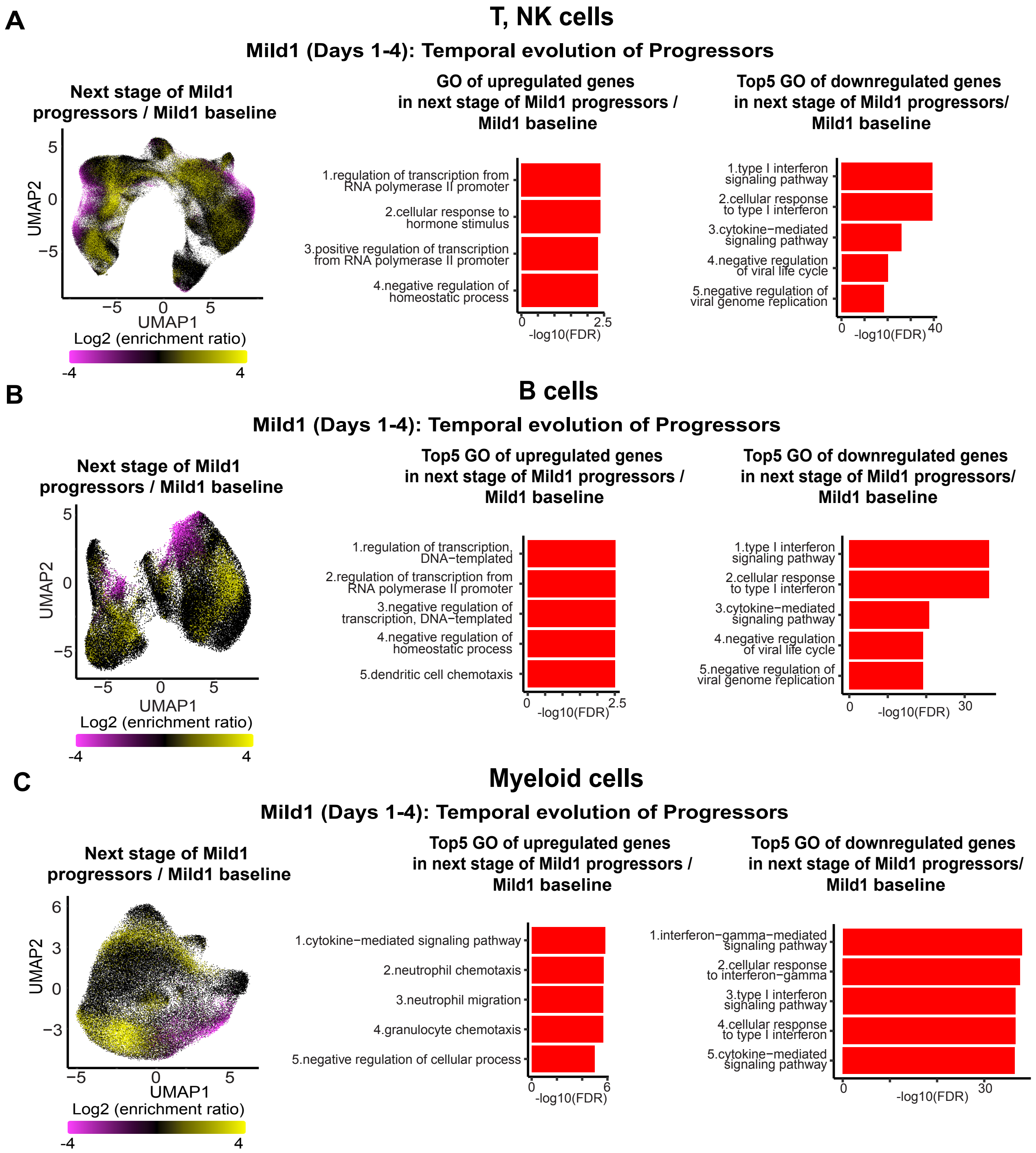


**Supplementary Figure 18. Temporal evolution of Mild1 Progressors (Days 1-4).** Temporal marker genes were identified in Mild1 Progressors (Days 1-4) across T, NK cells **(A)**, B cells **(B)** and myeloid cells **(C)**. UMAP plots on the left show the cell state enrichment fold changes between the next stage and baseline of Mild1 Progressors. Yellow color indicates cell states enriched in the next stage of Mild1 Progressors, while magenta denotes cell states depleted in the next stage of Mild1 Progressors. Bar plots in the middle show the top GO terms of upregulated genes in the next stage of Mild1 Progressors. Bar plots on the right show the top GO terms of downregulated genes in the next stage of Mild1 Progressors.

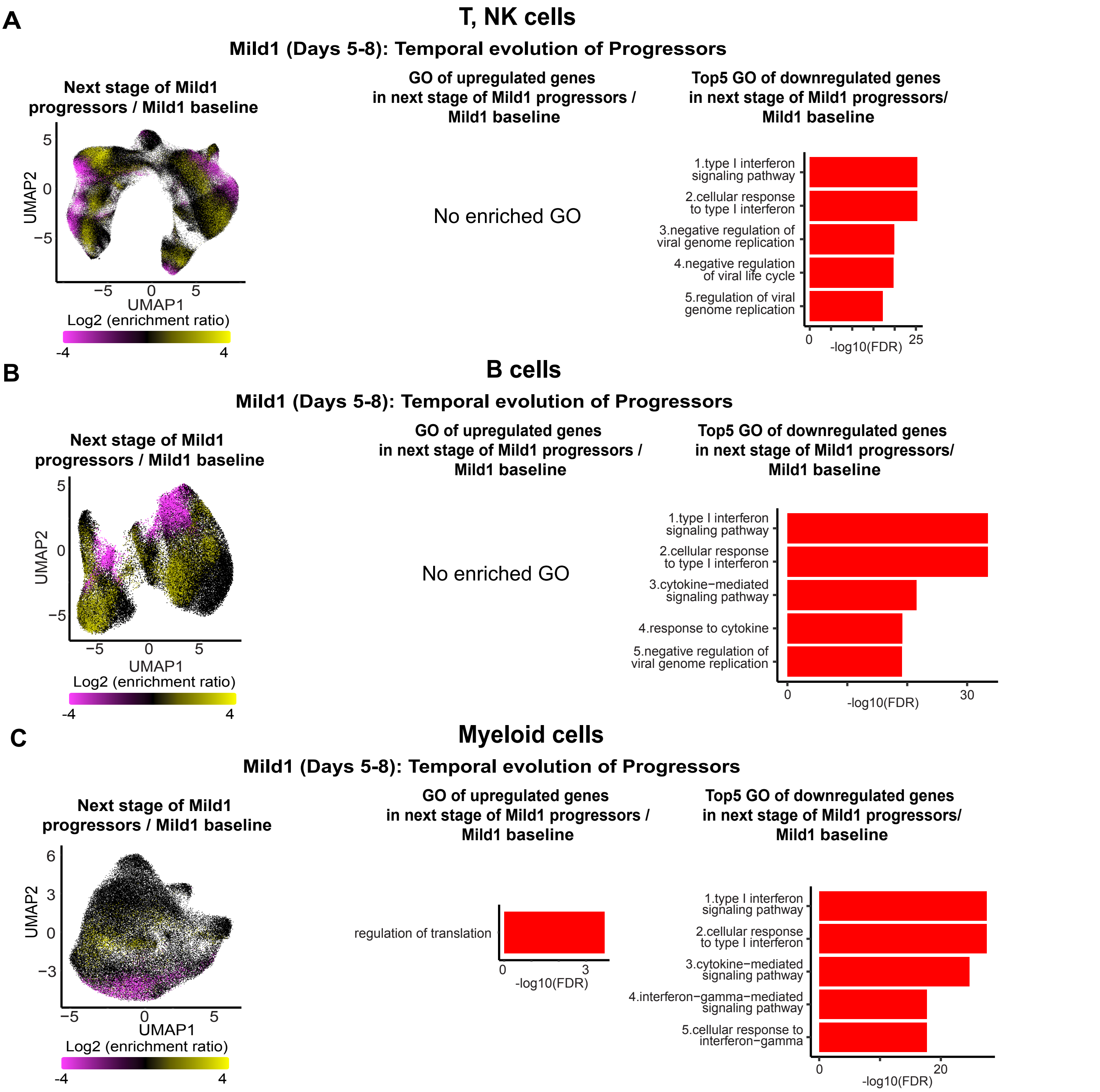


**Supplementary Figure 19. Temporal evolution of Mild1 Progressors (Days 5-8).** Temporal marker genes were identified in Mild1 Progressors (Days 5-8) across T, NK cells **(A)**, B cells **(B)** and myeloid cells **(C)**. UMAP plots on the left show the cell state enrichment fold changes between the next stage and baseline of Mild1 Progressors. Yellow color indicates cell states enriched in the next stage of Mild1 Progressors, while magenta denotes cell states depleted in the next stage of Mild1 Progressors. Bar plots in the middle show the top GO terms of upregulated genes in the next stage of Mild1 Progressors. Bar plots on the right show the top GO terms of downregulated genes in the next stage of Mild1 Progressors.

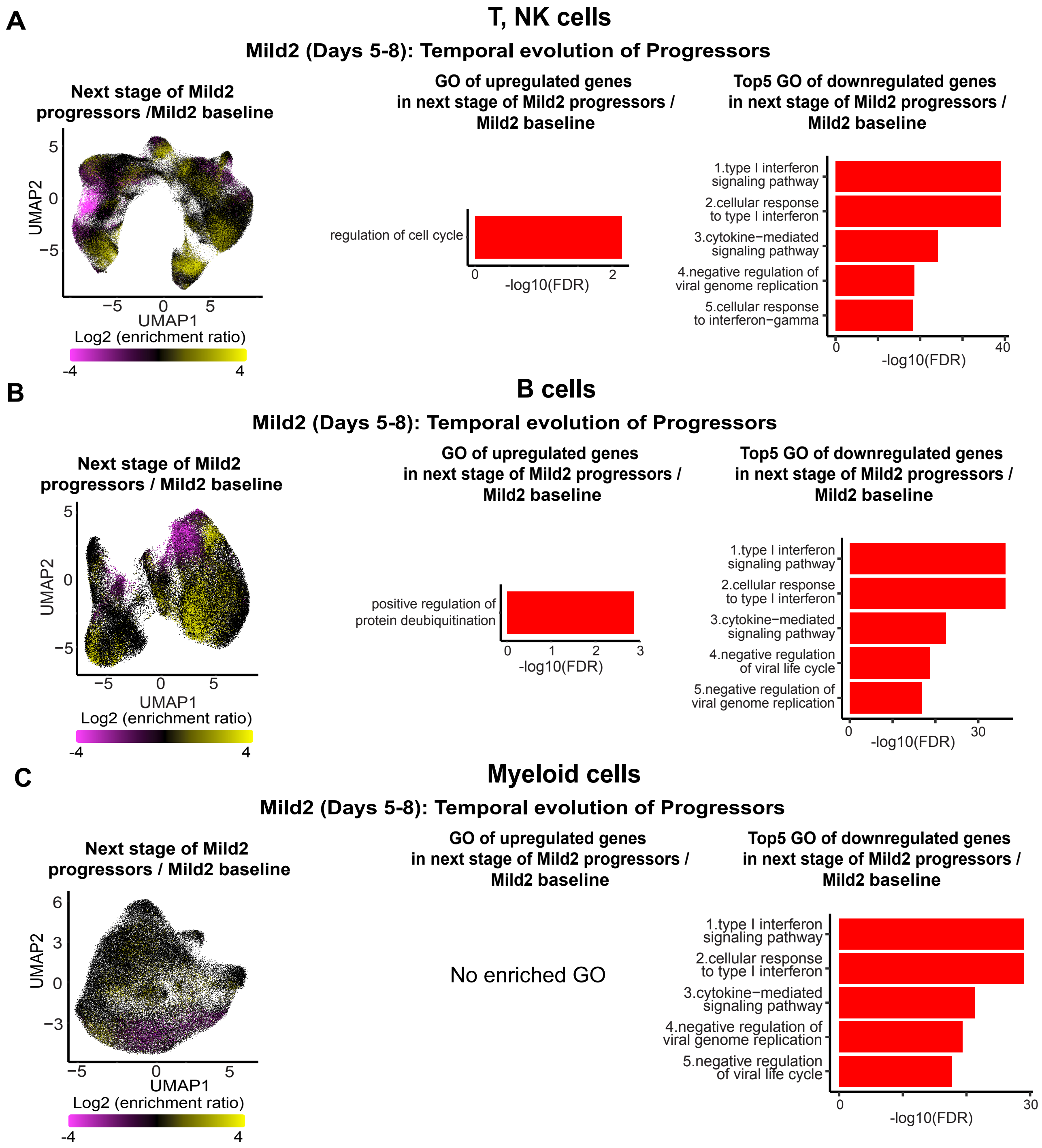


**Supplementary Figure 20. Temporal evolution of Mild2 Progressors (Days 5-8).** Temporal marker genes were identified in Mild2 Progressors (Days 5-8) across T, NK cells **(A)**, B cells **(B)** and myeloid cells **(C)**. UMAP plots on the left show the cell state enrichment fold changes between the next stage and baseline of Mild2 Progressors. Yellow color indicates cell states enriched in the next stage of Mild2 Progressors, while magenta denotes cell states depleted in the next stage of Mild2 Progressors. Bar plots in the middle show the top GO terms of upregulated genes in the next stage of Mild2 Progressors. Bar plots on the right show the top GO terms of downregulated genes in the next stage of Mild2 Progressors.

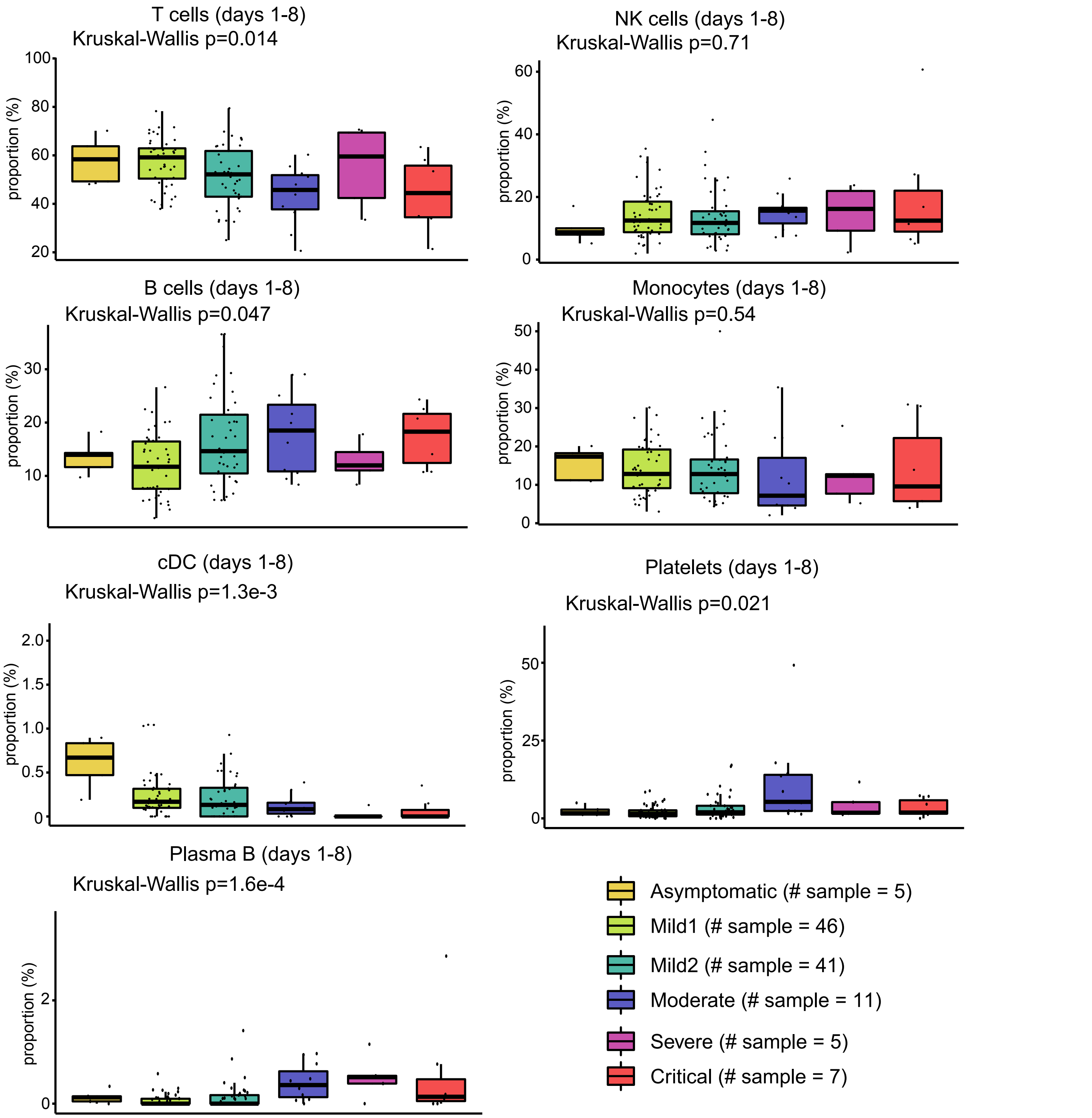


**Supplementary Figure 21. Cell type abundance across different severities for samples collected at Days 1- 8.** Box plots show cell type abundance across different severities for samples collected at Days 1-8. For each cell type, Kruskal-Wallis test was performed across severity. Box plots show the median (centre line), 25th and 75th percentile (lower and upper boundary), with 1.5x inter quartile range indicated by whiskers and outliers shown as individual data points.

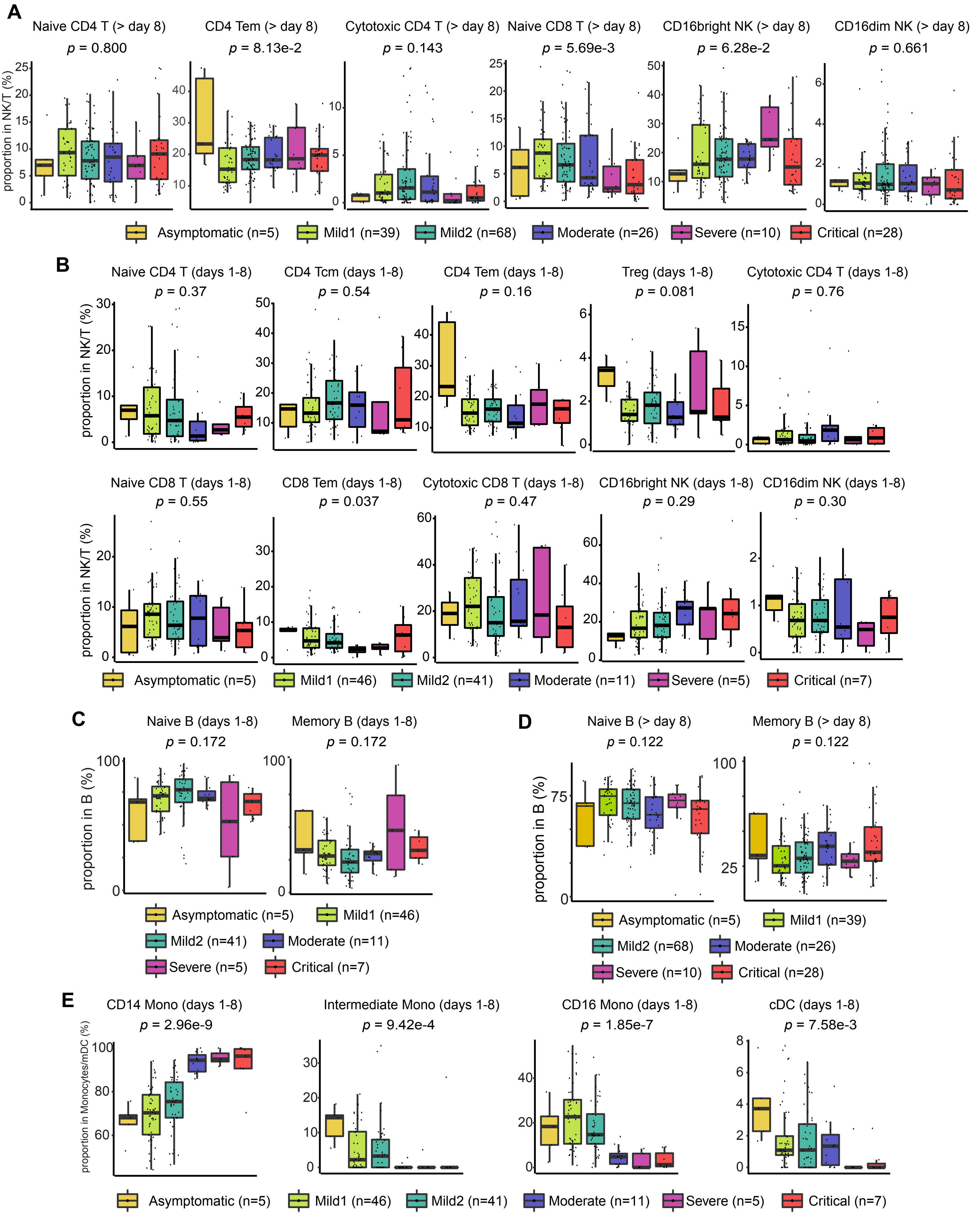


**Supplementary Figure 22.** Box plots show cell abundance across different severities in T, NK cell subtypes for samples collected after day 8 **(A)** and before day 8 **(B)**, B cell subtypes for samples collected before day 8 **(C)** and after day 8 **(D)**, as well as Myeloid cell subtypes for samples collected before day 8 **(E)**. For each cell type, Kruskal-Wallis test was performed across severity and its p-value is shown above each plot. Box plots show the median (centre line), 25th and 75th percentile (lower and upper boundary), with 1.5x inter quartile range indicated by whiskers and outliers shown as individual data points.

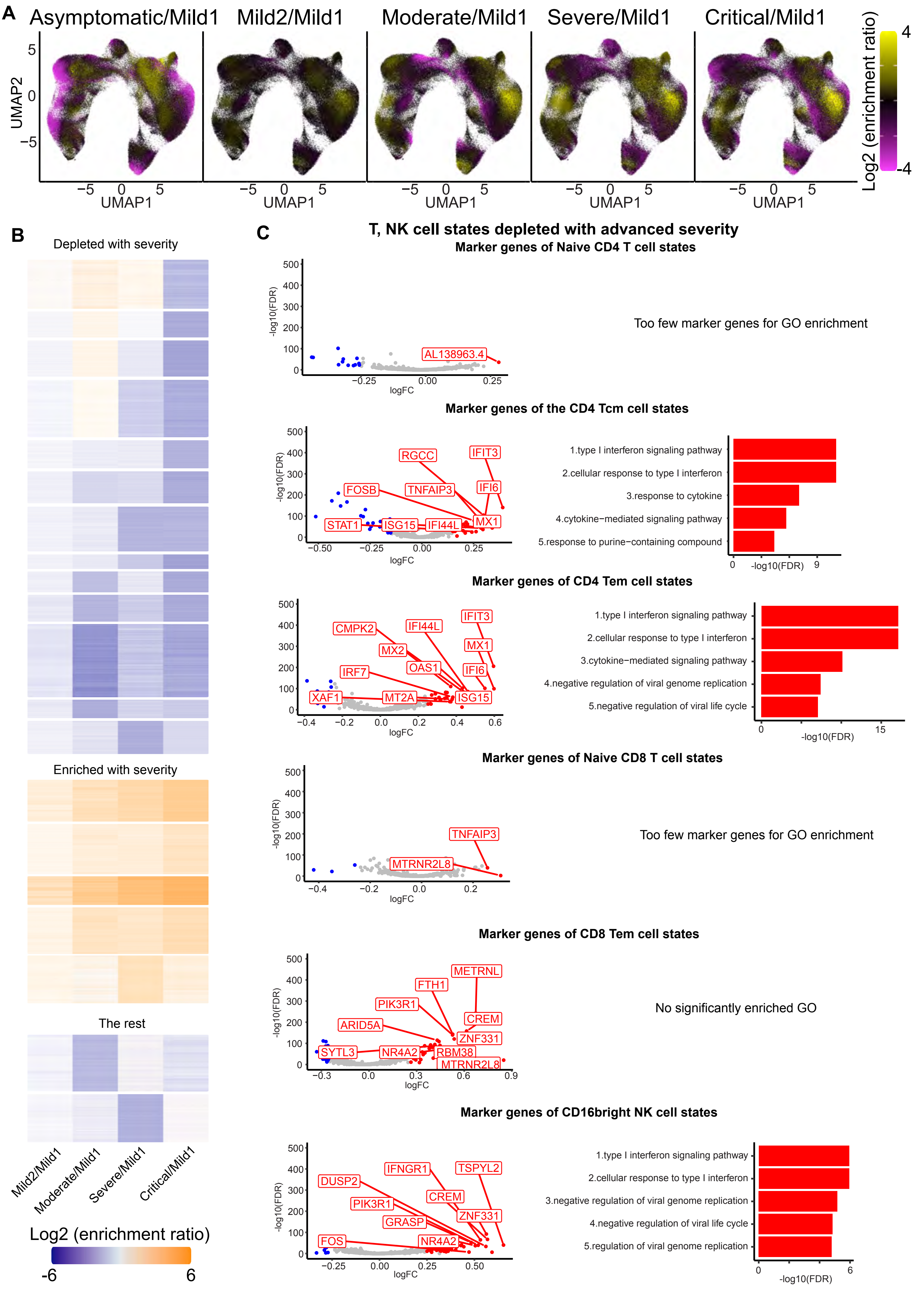
A
Asymptomatic/Mild1
Mild2/Mild1
Moderate/Mild1
Severe/Mild1
Critical/Mild1
UMAP2
UMAP1
Log2 (enrichment ratio)
B
Depleted with severity
Enriched with severity
The rest
Mild2/Mild1
Moderate/Mild1
Severe/Mild1
Critical/Mild1
Log2 (enrichment ratio)
-6
6
C
T, NK cell states depleted with advanced severity
Marker genes of Naive CD4 T cell states
AL138963.4
Too few marker genes for GO enrichment
Marker genes of the CD4 Tcm cell states
RGCC
IFIT3
FOSB
TNFAIP3
IFI6
STAT1
ISG15
IFI44L
MX1
1.type I interferon signaling pathway
2.cellular response to type I interferon
3.response to cytokine
4.cytokine−mediated signaling pathway
5.response to purine−containing compound
-log10(FDR)
Marker genes of CD4 Tem cell states
IFIT3
CMPK2
IFI44L
MX1
MX2
OAS1
IRF7
IFI6
XAF1
MT2A
ISG15
1.type I interferon signaling pathway
2.cellular response to type I interferon
3.cytokine−mediated signaling pathway
4.negative regulation of viral genome replication
5.negative regulation of viral life cycle
Marker genes of Naive CD8 T cell states
TNFAIP3
MTRNR2L8
Too few marker genes for GO enrichment
Marker genes of CD8 Tem cell states
METRNL
FTH1
PIK3R1
CREM
ARID5A
ZNF331
SYTL3
NR4A2
RBM38
MTRNR2L8
No significantly enriched GO
Marker genes of CD16bright NK cell states
IFNGR1
TSPYL2
DUSP2
CREM
PIK3R1
ZNF331
GRASP
FOS
NR4A2
1.type I interferon signaling pathway
2.cellular response to type I interferon
3.negative regulation of viral genome replication
4.negative regulation of viral life cycle
5.regulation of viral genome replication
logFC
-log10(FDR)

**Supplementary Figure 23. Cell state enrichments across severity in T, NK cells. A)** The UMAP plots show the cell-state enrichment fold changes between each severity and Mild1 (reference). Yellow color denotes cell states enriched with advanced severity, while magenta indicates cell states depleted with advanced severity. **B)** Heatmaps show the clusters of cell-state enrichment fold changes between each severity and Mild1. If the Pearson correlation for a cluster is greater than 0.5, it is considered enriched with severity. If the Pearson correlation for a cluster is less than -0.5, it is considered depleted with severity. If the Pearson correlation for a cluster is between -0.5 and 0.5, it is grouped under the rest. **C)** Volcano plots show the up/down-regulated genes between cell states depleted across severity and the remaining cell states in each cell subtype. Bar plots show the enriched GO terms associated with upregulated genes in cell states depleted with severity for each cell subtype.

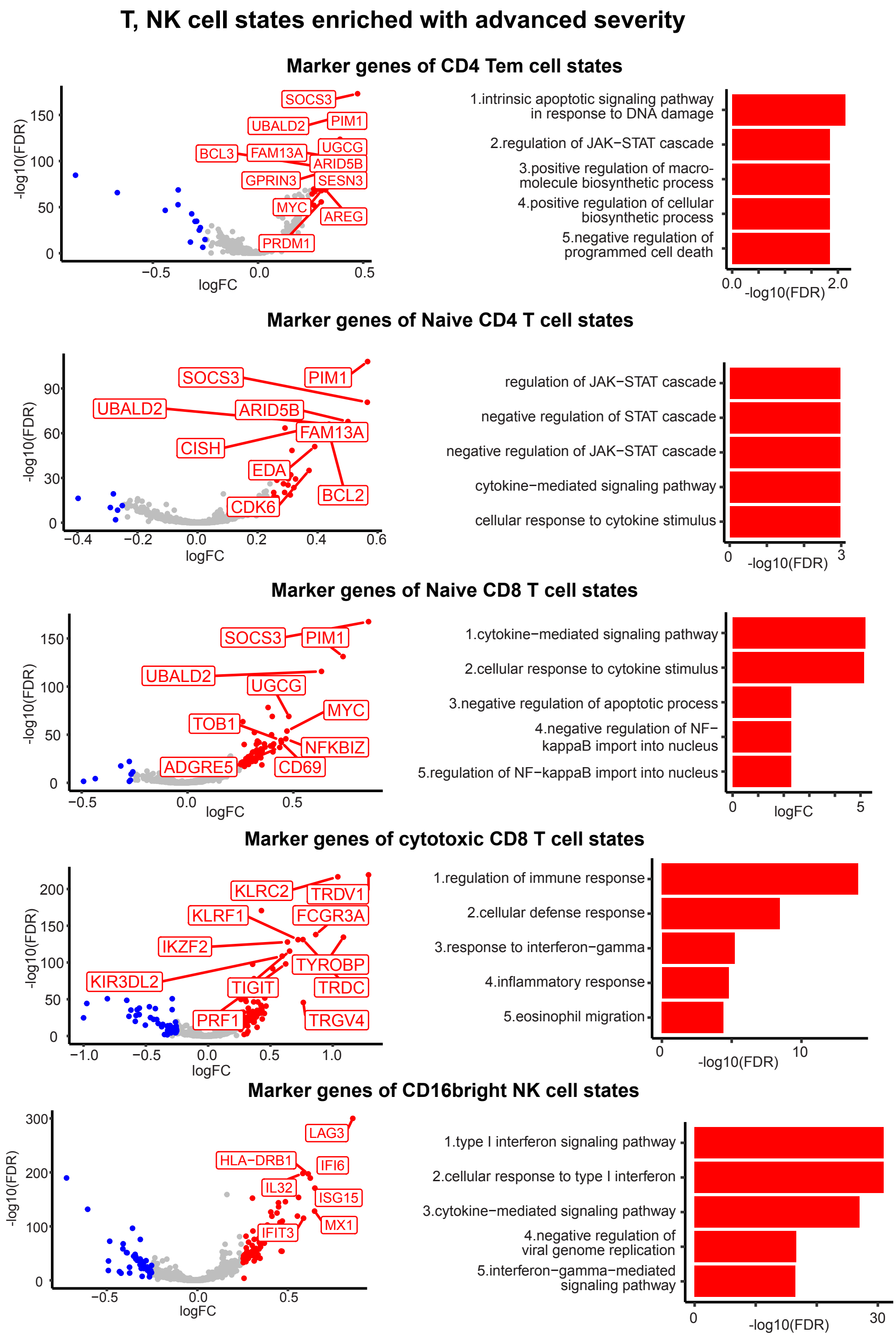


**Supplementary Figure 24. Marker genes of T, NK cell states enriched with advanced severity.** Volcano plots show the up/down-regulated genes between cell states enriched in advanced severity and the remaining cell states in each cell subtype. Bar plots show the enriched GO terms associated with upregulated genes in cell states enriched with severity for each cell subtype.

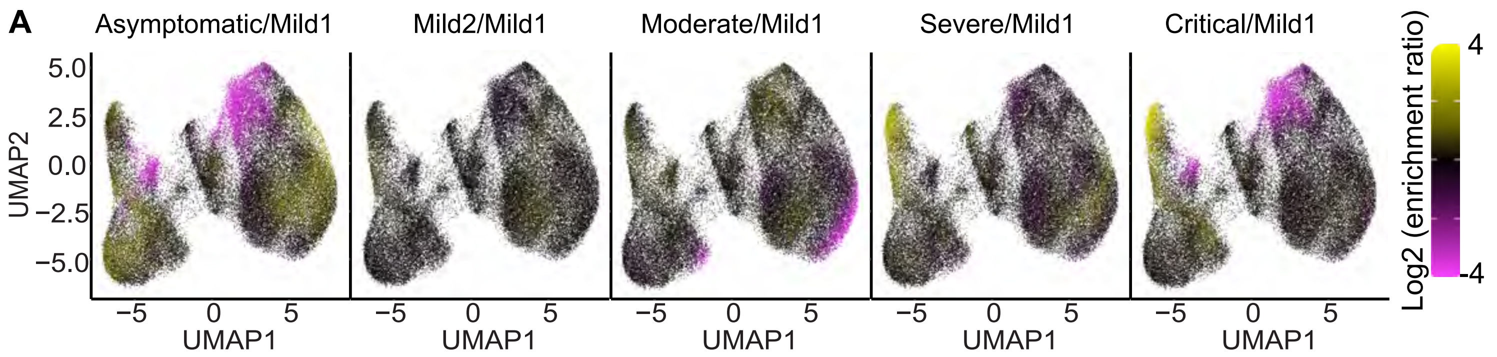


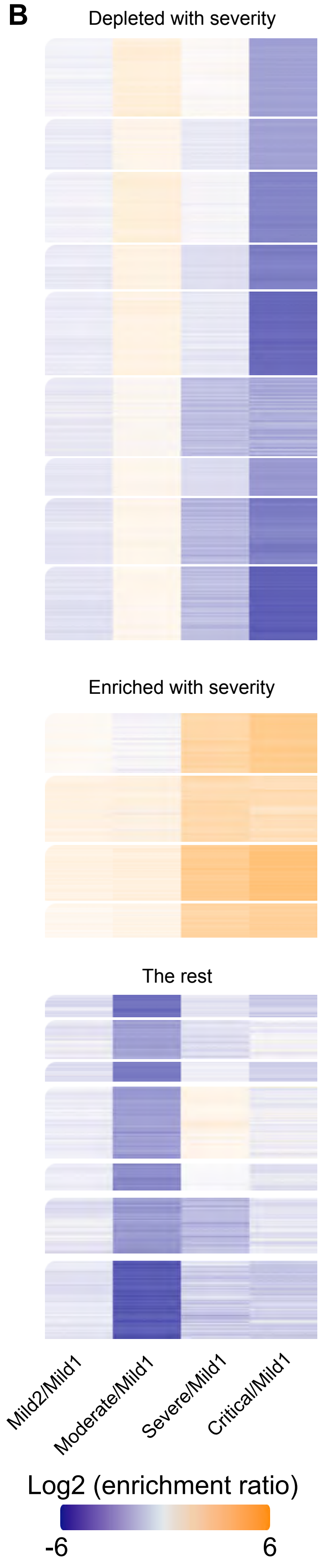


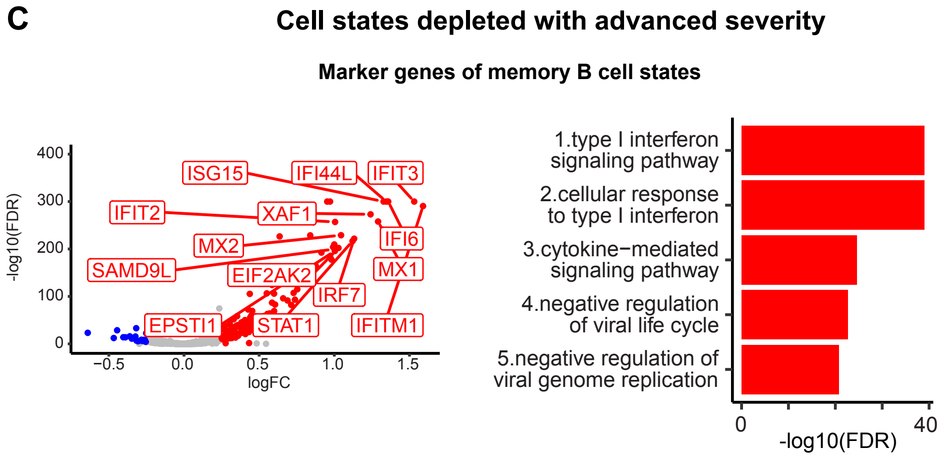


**Supplementary Figure 25. Cell state enrichment across severity in B cells. A)**The UMAP plots show the cell-state enrichment fold changes between each severity and Mild1 (reference). Yellow color denotes cell states enriched with advanced severity, while magenta indicates cell states depleted with advanced severity. **B)** Heatmaps show the clusters of cell-state enrichment fold changes between each severity and Mild1. If the Pearson correlation for a cluster is greater than 0.5, it is considered enriched with severity. If the Pearson correlation for a cluster is less than -0.5, it is considered depleted with severity. If the Pearson correlation for a cluster is between -0.5 and 0.5, it is grouped under the rest. **C)** Volcano plots show the up/down-regulated genes between cell states depleted across severity and the remaining cell states in memory B cells. Bar plots show the enriched GO terms associated with up-regulated genes in cell states depleted across severity for memory B cells.

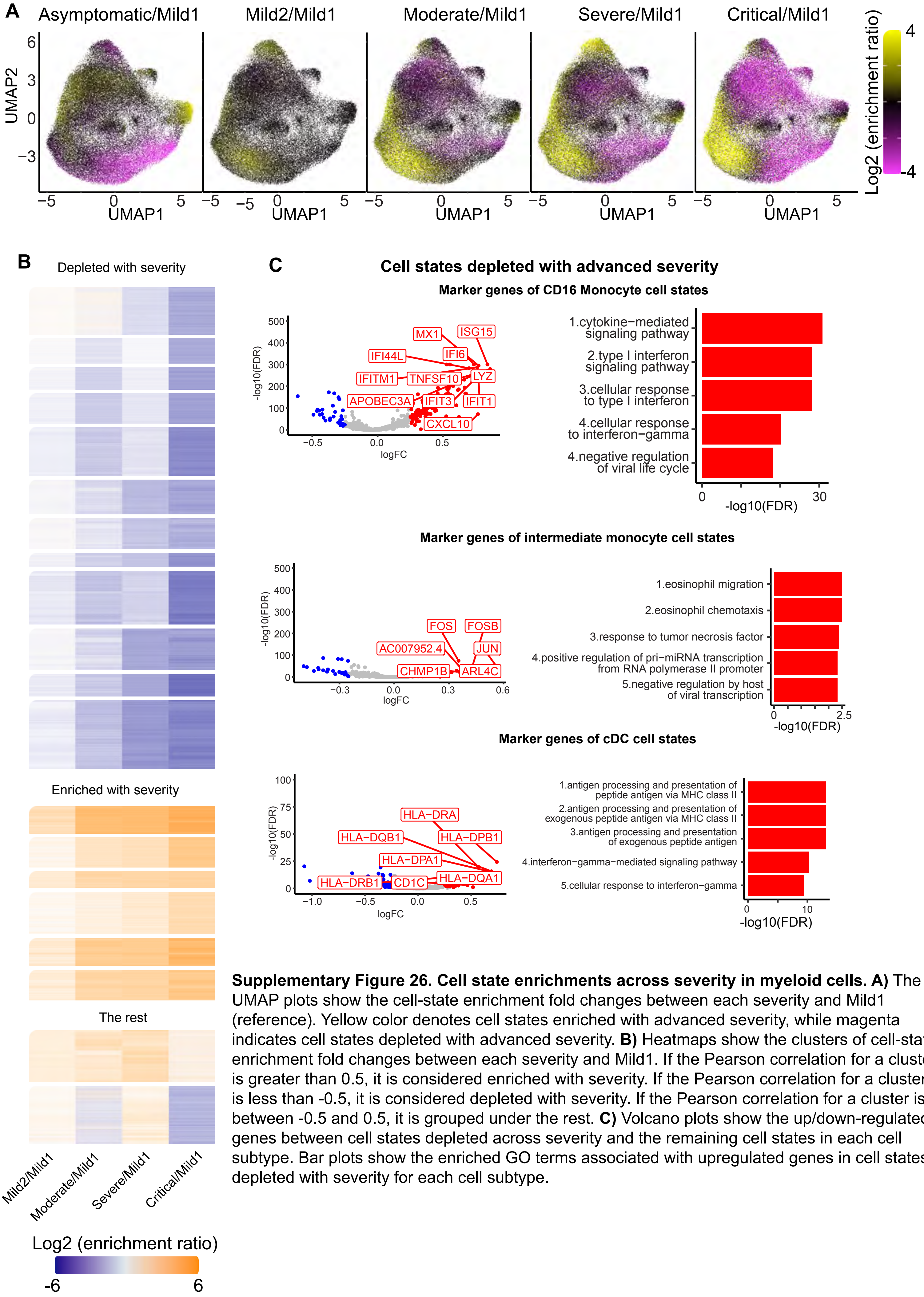


**Supplementary Figure 26. Cell state enrichments across severity in myeloid cells. A)** The UMAP plots show the cell-state enrichment fold changes between each severity and Mild1 (reference). Yellow color denotes cell states enriched with advanced severity, while magenta indicates cell states depleted with advanced severity. **B)** Heatmaps show the clusters of cell-state enrichment fold changes between each severity and Mild1. If the Pearson correlation for a cluster is greater than 0.5, it is considered enriched with severity. If the Pearson correlation for a cluster is less than -0.5, it is considered depleted with severity. If the Pearson correlation for a cluster is between -0.5 and 0.5, it is grouped under the rest. **C)** Volcano plots show the up/down-regulated genes between cell states depleted across severity and the remaining cell states in each cell subtype. Bar plots show the enriched GO terms associated with upregulated genes in cell states depleted with severity for each cell subtype.

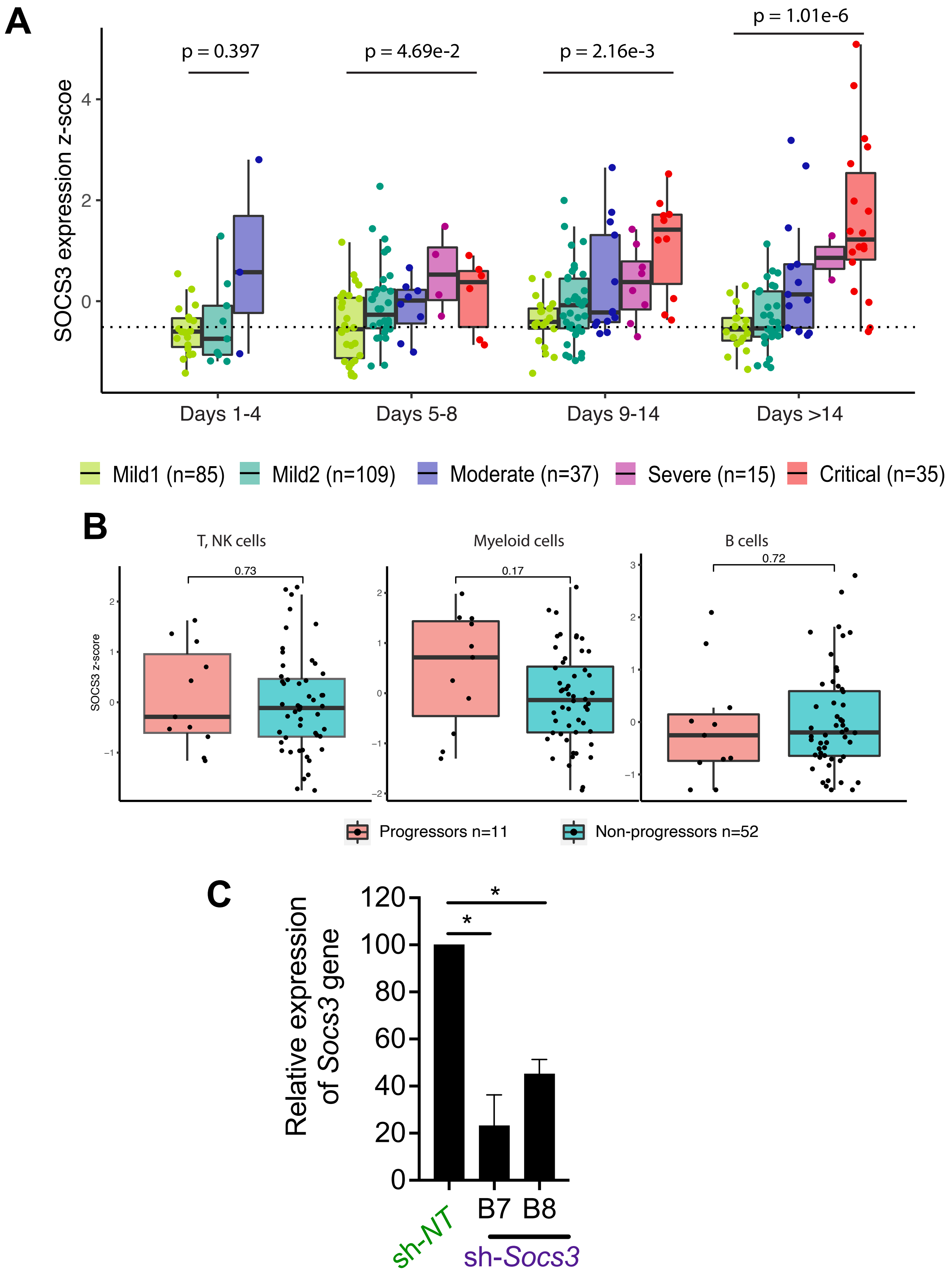


**Supplementary Figure 27: A)** SOCS3 gene expression (pseudobulk) among all T, NK cells in each sample. Samples are grouped by disease severity and duration. Horizontal dashed line: average SOCS3 z-score of the 5 asymptomatic (Asy) samples. P-values: Kruskal-Wallis test. **B)** Left panel: boxplot of the z-score of pseudobulk (averaged across T, NK cells in one sample) SOCS3 expression in Progressors (n=11) vs Non-Progressors (n=52). P-value: Student's t-test (two-sided). Middle, right panels: similar boxplots for myeloid cells and B cells, respectively. Box plots show the median (centre line), 25th and 75th percentile (lower and upper boundary), with 1.5x inter quartile range indicated by whiskers and outliers shown as individual data points. **C)** Socs3 expression was suppressed in HEK-Ace2 cells by shRNA mediated knockdown (with two distinct clones B7 and B8 of sh-Socs3). Non-targeting shRNA (sh-NT) was used as a control. After 24 hrs of transfection by shRNA, RNA was isolated and expression of Socs3 (relative to house -keeping gene, see Methods) was assessed by qRT-PCR. Error bars represent SD of 3 technical replicates. P-values t-test. *, P<0.05.

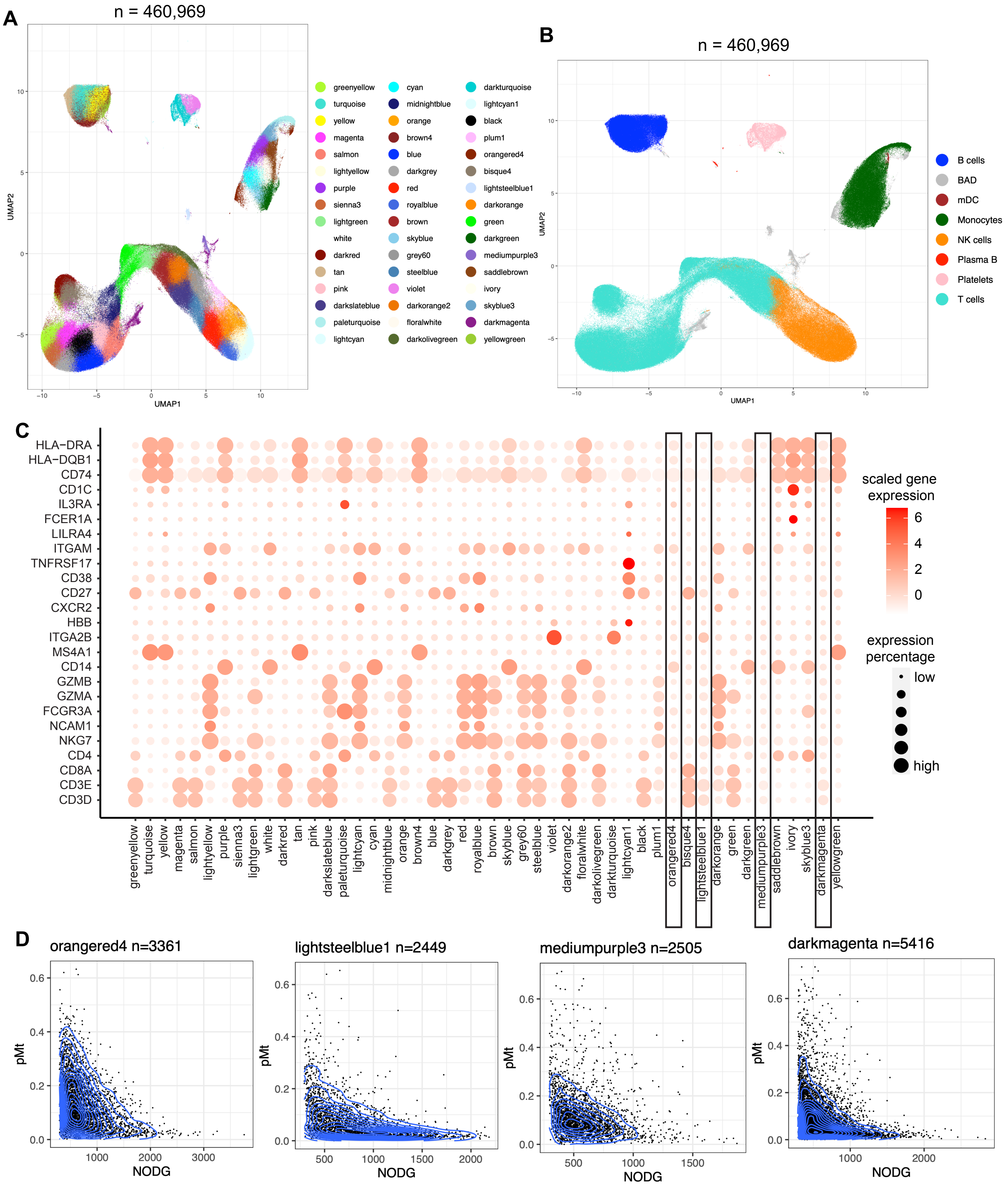


**Supplementary Figure 28. A)** UMAP plot shows the PBMC cell clusters by RCA2 after doublet removal but before QC. **B)** UMAP plot represents the annotated cell clusters by RCA2 after doublet removal but before QC. **C)** Bubble plot shows the expression profiles of well-documented PBMC marker genes across different clusters shown in panel **A**. The clusters in the black boxes are the low quality cells (annotated as “BAD” in panel **B**). **D)** 2D density plots represent the number of detected genes (NODG) and mitochondrial percentage (pMt) for the cell clusters in the black boxes in panel **C**.

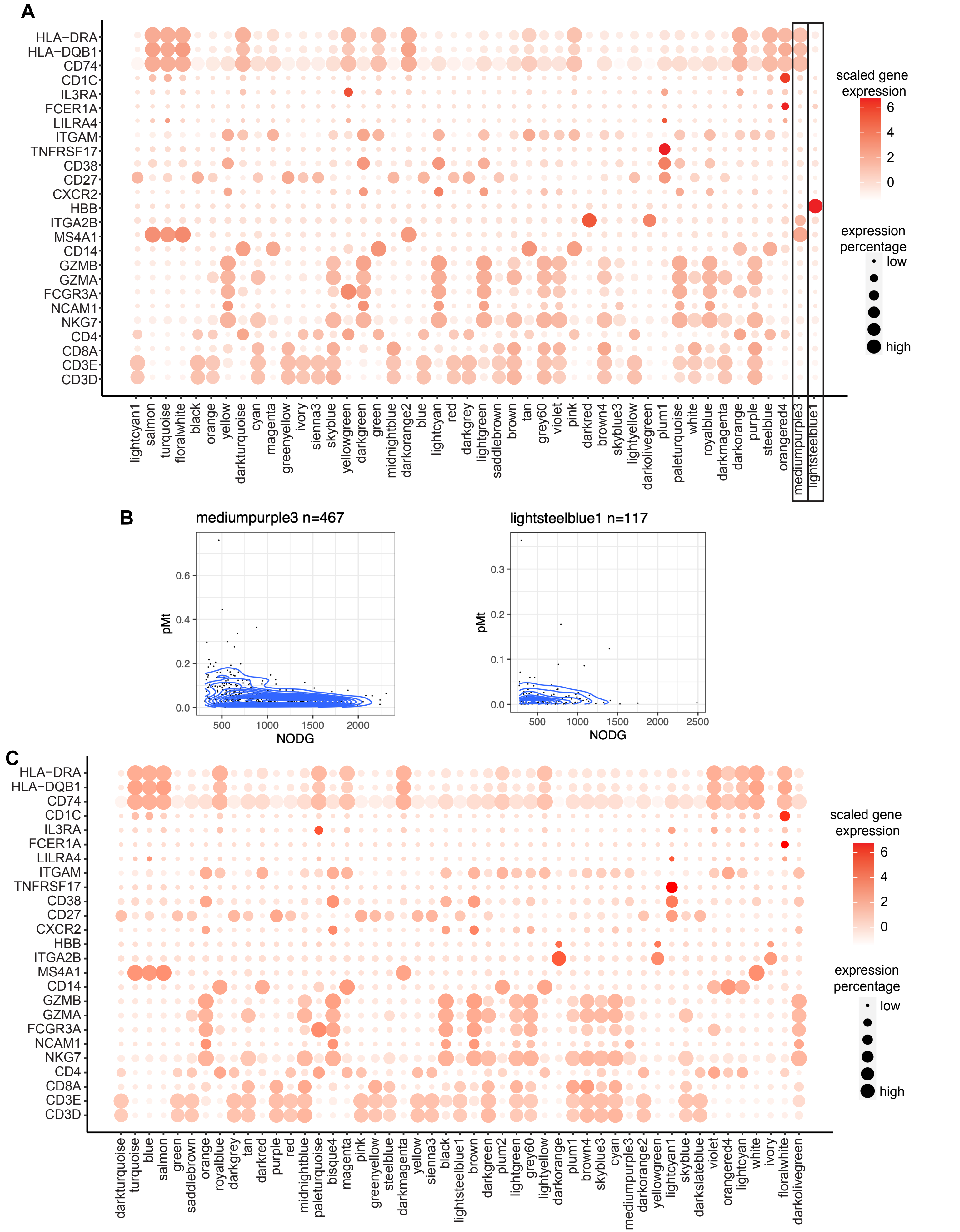


**Supplementary Figure 29. Marker gene profiling of single cell clusters. A)** After removing clusters with low quality cells, the remaining cells were re-clustered using RCA2. Bubble plot shows the expression profiles of well-documented PBMC marker genes across different clusters after re-clustering. **B**) 2D density plots represent the number of detected genes (NODG) and mitochondrial percentage (pMt) for the cell clusters in the black boxes in panel **A**. **C)** After discarding clusters highlighted in black boxes in panel **A**, the remaning cells were re-clustered using RCA2. Bubble plot shows the expression profiles of well-documented PBMC marker genes across different clusters after re-clustering.

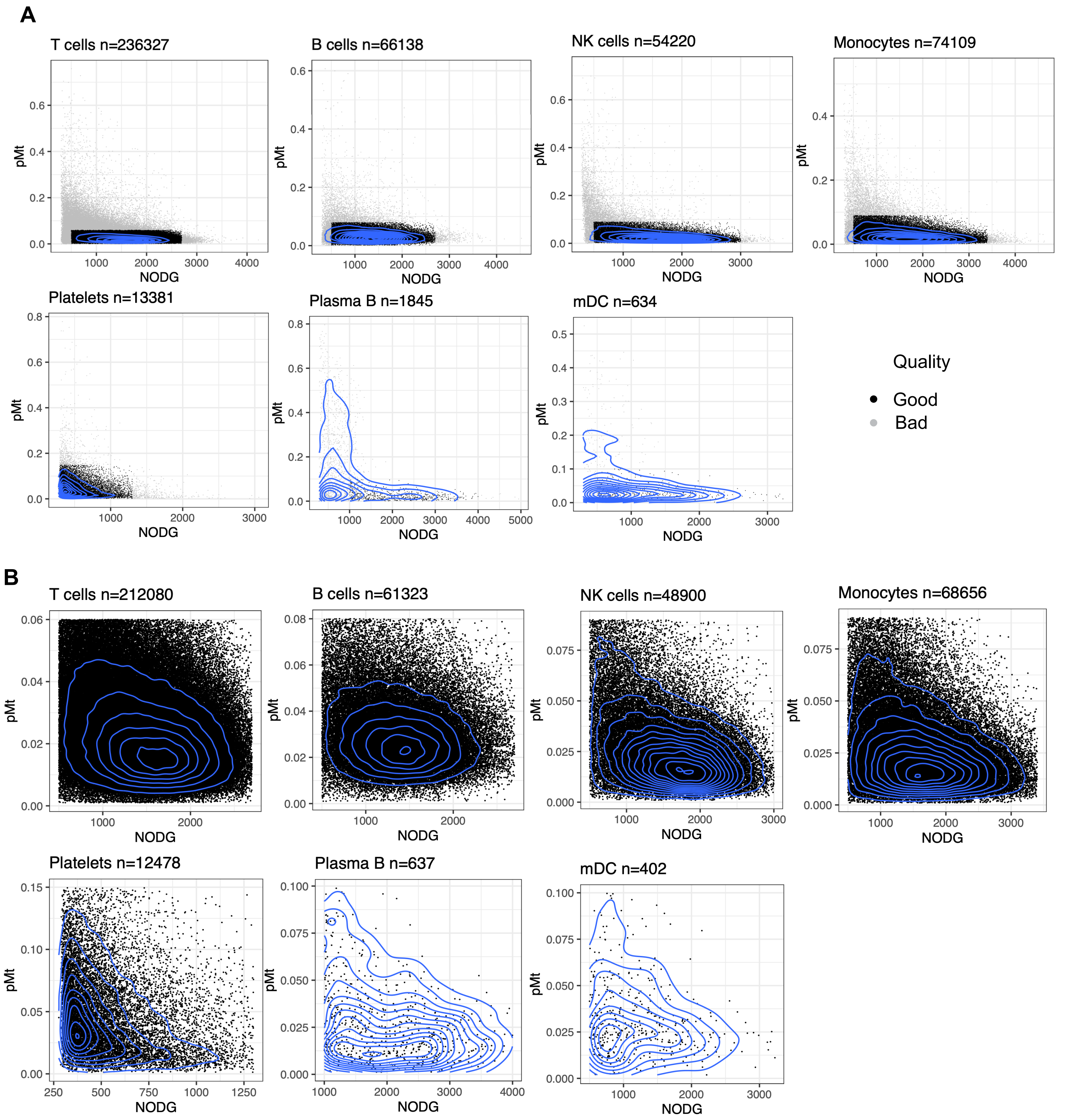


**Supplementary Figure 30. Single cell analysis pipeline: QC criteria. A)** The 2D density plots show the number of detected gene (NODG) and mitochondrial percentage (pMt) across different major cell types before QC. Black dots indicate cells to be retained, while grey dots represent cells to be discarded. **B)** The 2D density plots show the NODG and pMt across different major cell types after QC.

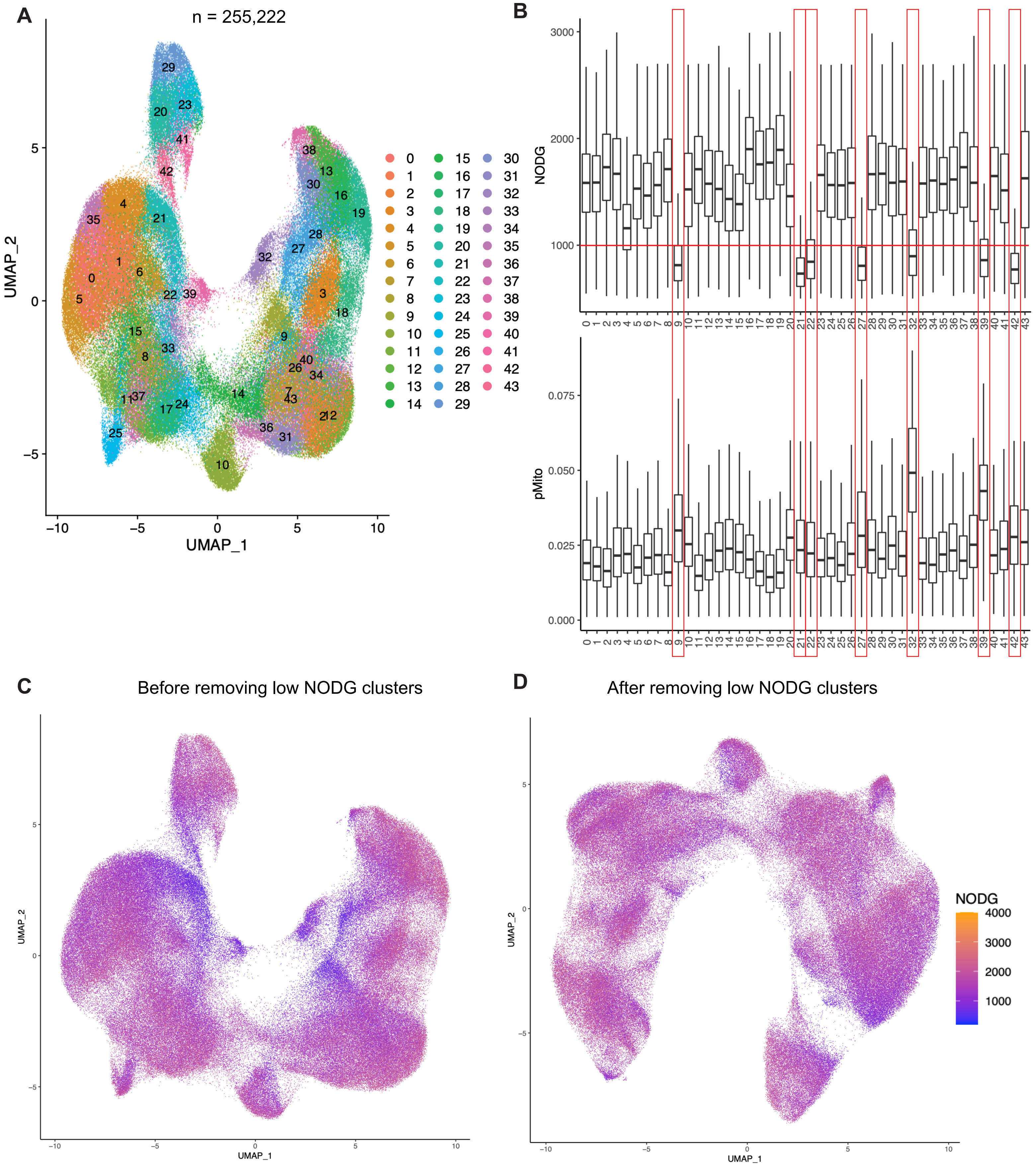


**Supplementary Figure 31. Sub-clustering of T and NK cells. A)** The integrated UMAP shows the sub-clustering results in T, NK cells. **B)** The box plots illustrate the number of detected genes (NODG, upper) and mitochondrial percentage (pMito, lower) across all clusters in panel. Box plots show the median (centre line), 25th and 75th percentile (lower and upper boundary), with 1.5x inter quartile range indicated by whiskers and outliers shown as individual data points. **A. C)** The integrated UMAP in T, NK cells shows NODG profiles across all cells before removing low NODG clusters highlighted in red boxes in panel **B. D)** The integrated UMAP in T, NK cells shows NODG profiles across all cells after removing low NODG clusters highlighted in red boxes in panel **B.**

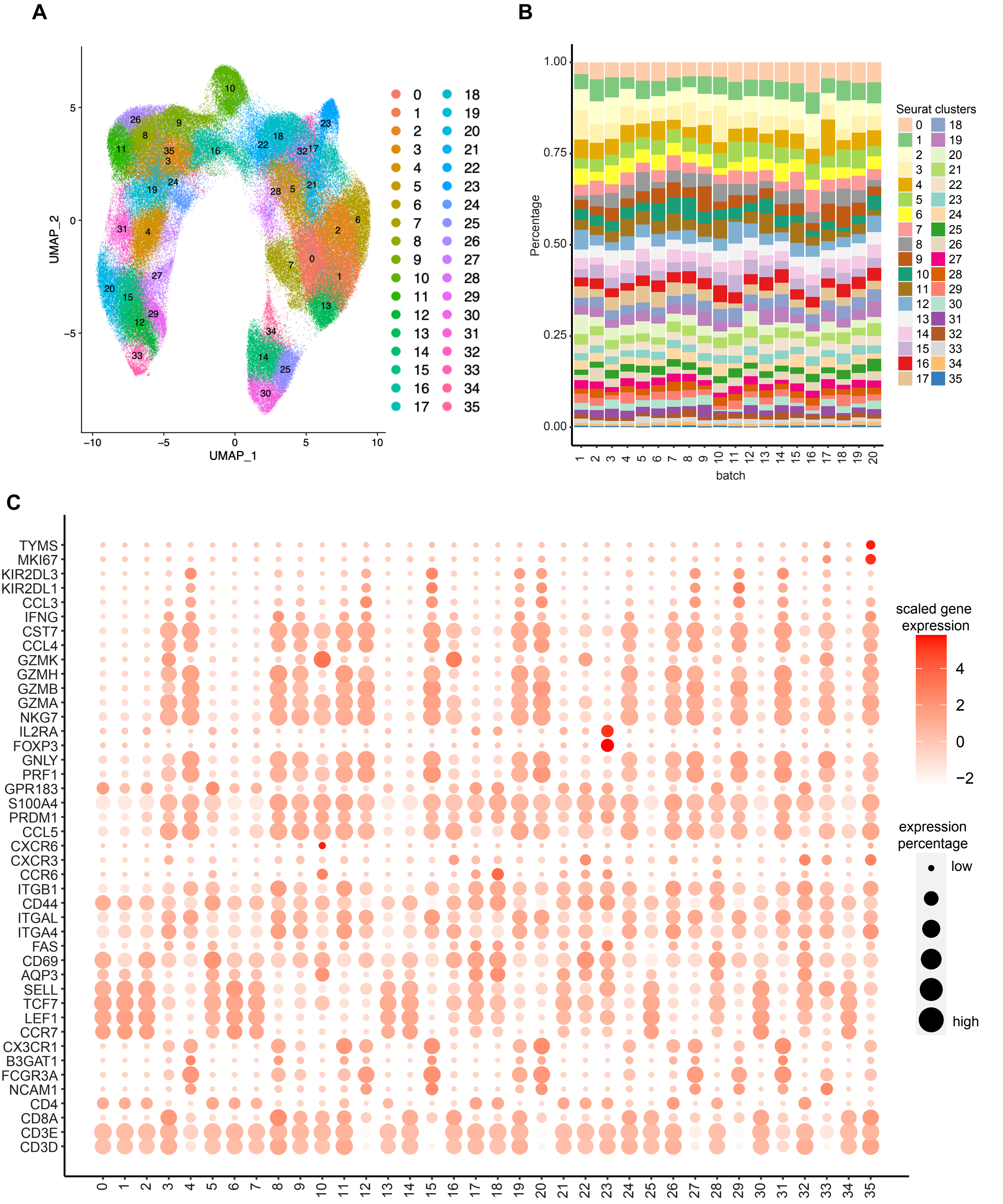


**Supplementary Figure 32. Marker gene profile of T, NK sub-clusters. A)** The integrated UMAP shows the re-clustering results in T, NK cells after removing clusters with low NODG. **B)** The bar plot illustrates the distributions of 36 sub-clusters in T,NK cells across all 20 experiment batches to check batch effects. **C)** The bubble plot shows the expression profiles of well-documented T, NK marker genes across all 36 sub-clusters.

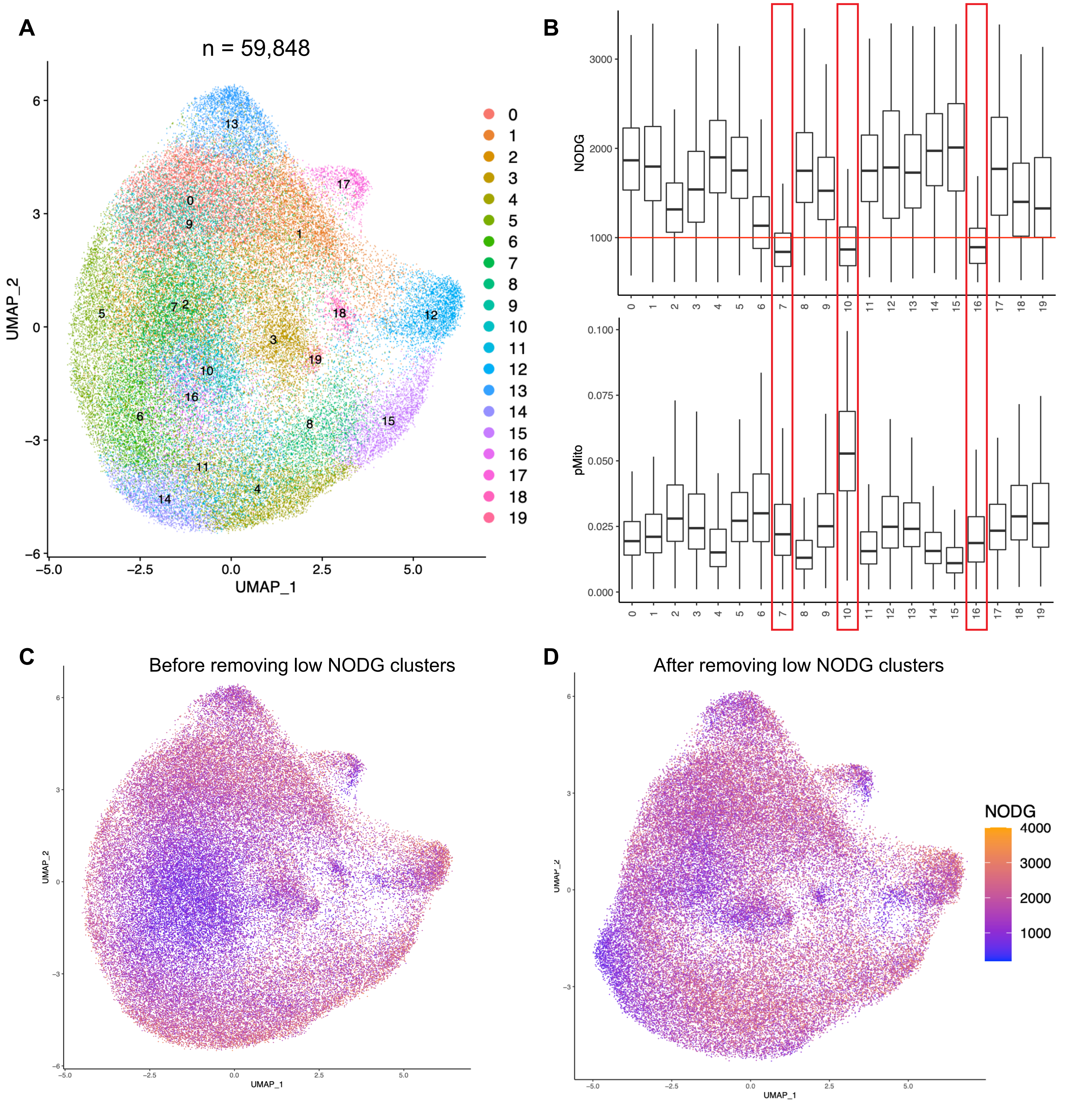


**Supplementary Figure 33. Sub-clustering of myeloid cells. A)** The integrated UMAP shows the sub-clustering results in myeloid cells. **B)** The box plots illustrate the number of detected genes (NODG, upper) and mitochondrial percentage (pMito, lower) across all clusters in panel. Box plots show the median (centre line), 25th and 75th percentile (lower and upper boundary), with 1.5x inter quartile range indicated by whiskers and outliers shown as individual data points. **A. C)** The integrated UMAP in myeloid cells shows NODG profiles across all cells before removing low NODG clusters highlighted in red boxes in panel **B. D)** The integrated UMAP in myeloid cells shows NODG profiles across all cells after removing low NODG clusters highlighted in red boxes in panel **B**.

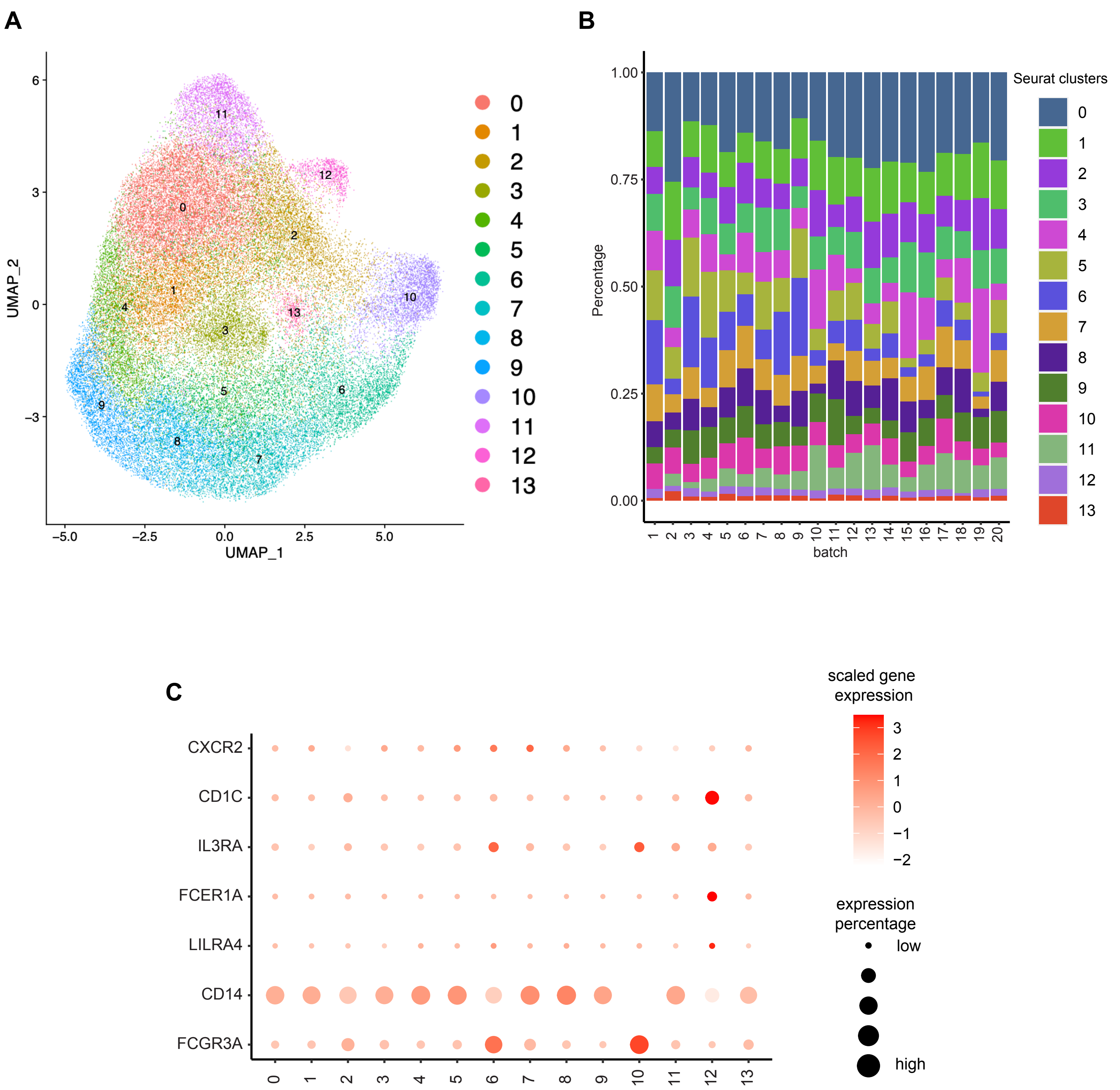


**Supplementary Figure 34. Marker gene profile of myeloid cell sub-clusters. A)** The integrated UMAP shows the re-clustering results in myeloid cells after removing clusters with low NODG. **B)** The bar plot illustrates the distributions of 14 sub-clusters in myeloid cells across all 20 experiment batches to check batch effects. **C)** The bubble plot shows the expression profiles of well-documented meyloid marker genes across all 14 sub-clusters.

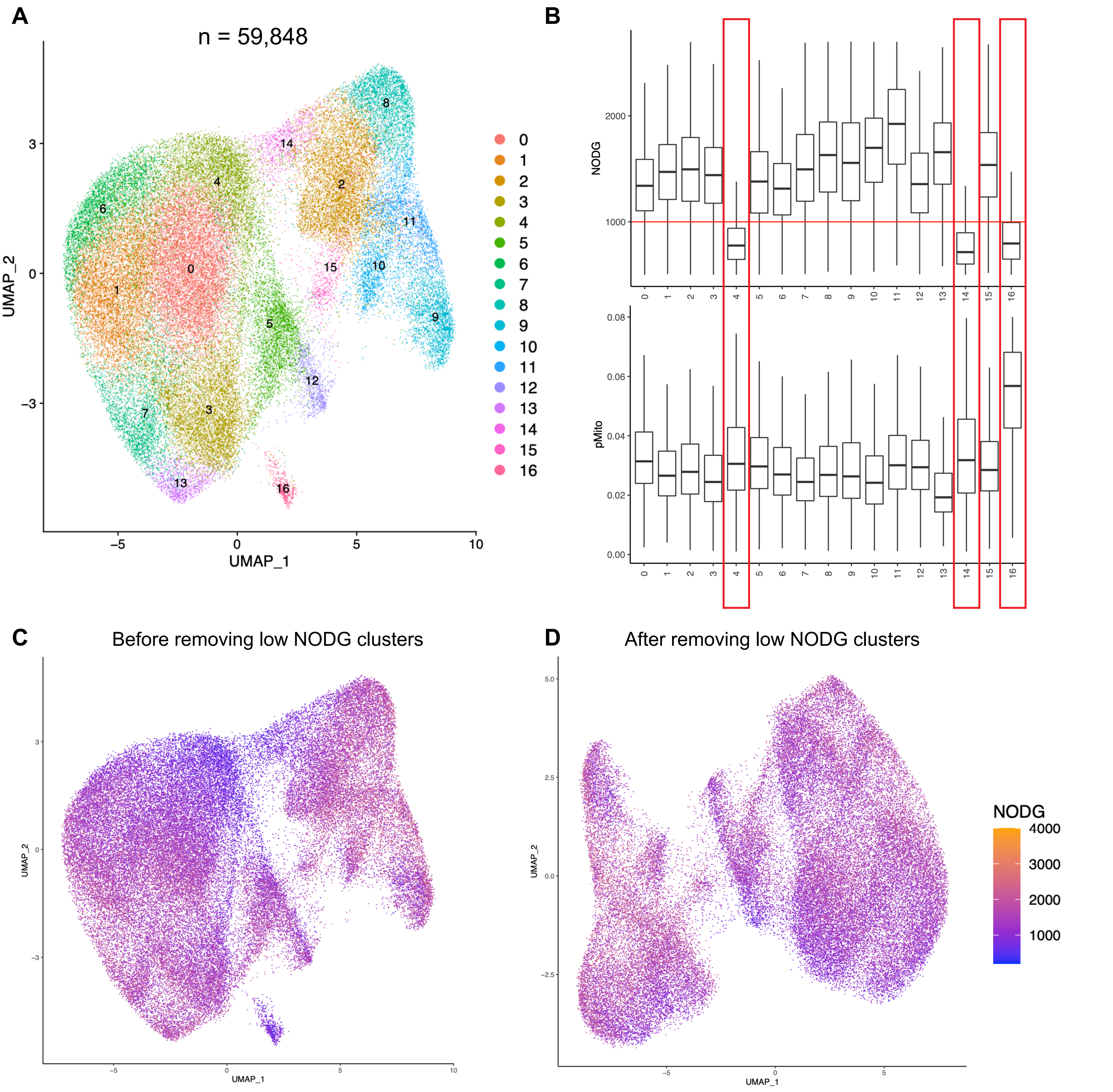


**Supplementary Figure 35. Sub-clustering of B cells. A)** The integrated UMAP shows the sub-clustering results in B cells. **B)** The box plots illustrate the number of detected genes (NODG, upper) and mitochondrial percentage (pMito, lower) across all clusters in panel. Box plots show the median (centre line), 25th and 75th percentile (lower and upper boundary), with 1.5x inter quartile range indicated by whiskers and outliers shown as individual data points.  **A. C)** The integrated UMAP in B cells shows NODG profiles across all cells before removing low NODG clusters highlighted in red boxes in panel **B. D)** The integrated UMAP in B cells shows NODG profiles across all cells after removing low NODG clusters high-lighted in red boxes in panel **B**.

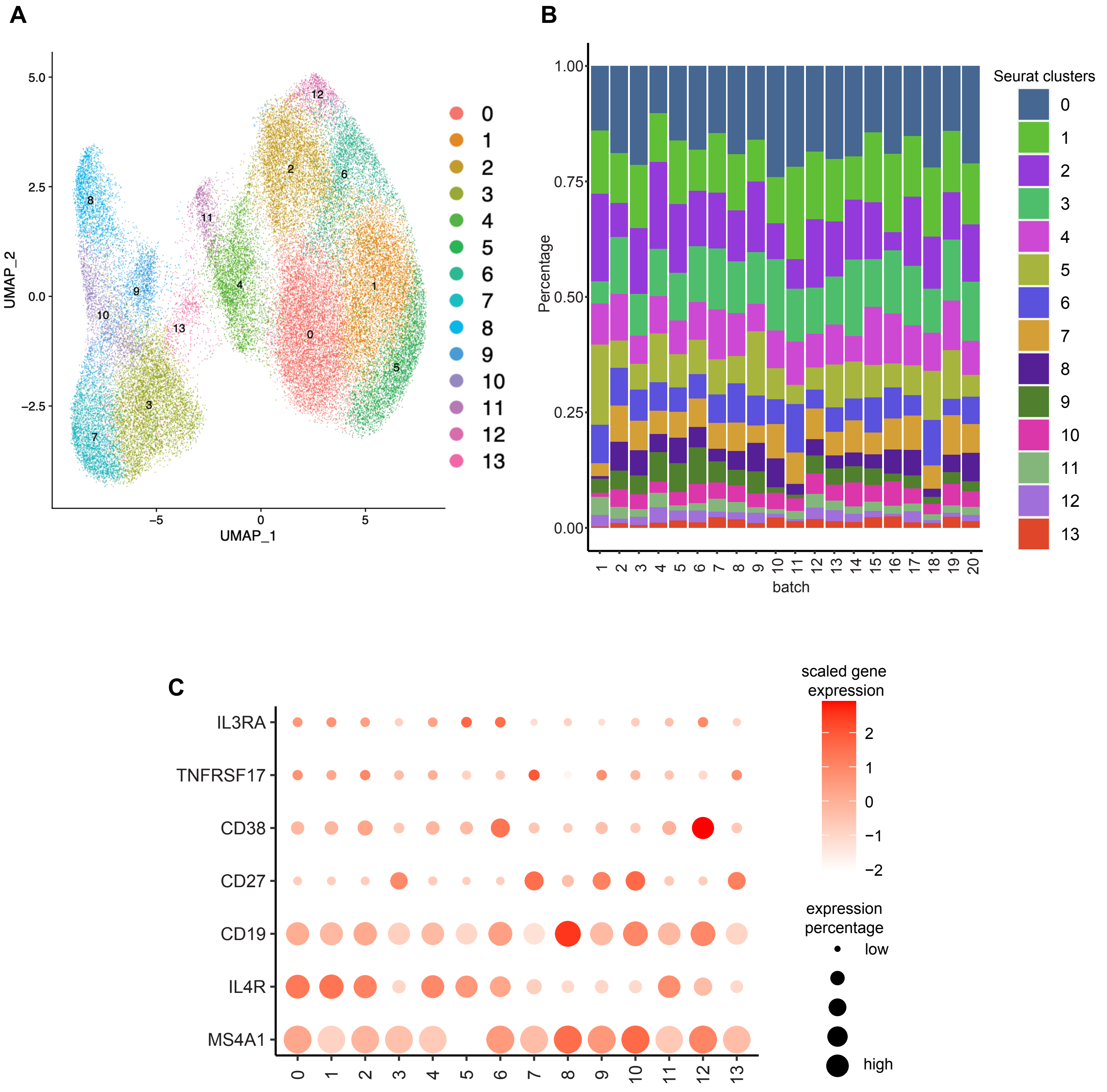


**Supplementary Figure 36. Marker gene profile of B cell sub-clusters. A)** The integrated UMAP shows the re-clustering results in B cells after removing clusters with low NODG. **B)** The bar plot illustrates the distributions of 14 sub-clusters in B cells across all 20 experiment batches to check batch effects. **C)** The bubble plot shows the expression profiles of well-documented B cell marker genes across all 14 sub-clusters.

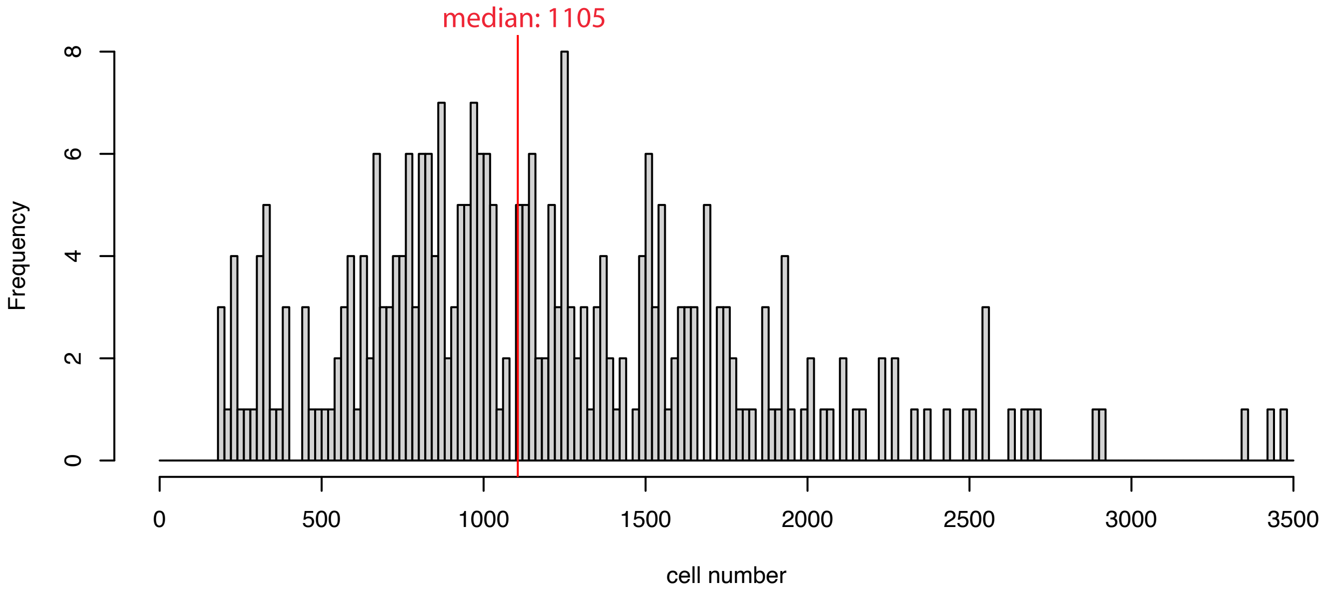


**Supplementary Figure 37.** Histogram of number of high-quality (post-QC) cells per sample.
