# Supplementary material for: Longitudinal single cell atlas identifies complex temporal relationship between type I interferon response and COVID-19 severity
Source: Nat Commun. 2024 Jan 18;15:567. doi: 10.1038/s41467-023-44524-0 (PMC10796319; doi:10.1038/s41467-023-44524-0)
Supplement: Supplementary file 3 — Description of Additional Supplementary Files [file 41467_2023_44524_MOESM3_ESM.pdf]

Description of Additional Supplementary Files

Supplementary Data 1 - Sample information

Supplementary Data 2 - Sample batching details

Supplementary Data 3 - Marker genes in prognostic and longitudinal analysis and pvalue in plasma protein analysis between progressors and nonprogressors

Supplementary Data 4 - GO terms of marker genes in prognostic and longitudinal analysis

Supplementary Data 5 - Marker genes in cross-sectional analysis

Supplementary Data 6 - GO terms and KEGG pathways of marker genes in cross-sectional analysis

Supplementary Data 7 - German cohort study and downsampling in cross-sectional analysis
