# Supplementary material for: Longitudinal single cell atlas identifies complex temporal relationship between type I interferon response and COVID-19 severity
Source: Nat Commun. 2024 Jan 18;15:567. doi: 10.1038/s41467-023-44524-0 (PMC10796319; doi:10.1038/s41467-023-44524-0)
Supplement: Supplementary file 11 — Reporting Summary [file 41467_2023_44524_MOESM11_ESM.pdf]

nature portfolio

Corresponding author(s): Amit Singhal and Shyam Prabhakar

Last updated by author(s): Nov 19, 2023

# Reporting Summary

Nature Portfolio wishes to improve the reproducibility of the work that we publish. This form provides structure for consistency and transparency in reporting. For further information on Nature Portfolio policies, see our Editorial Policies and the Editorial Policy Checklist.

## Statistics

For all statistical analyses, confirm that the following items are present in the figure legend, table legend, main text, or Methods section.

| n/a | Confirmed | |
|---|---|---|
| ☐ | ☒ | The exact sample size (*n*) for each experimental group/condition, given as a discrete number and unit of measurement |
| ☐ | ☒ | A statement on whether measurements were taken from distinct samples or whether the same sample was measured repeatedly |
| ☐ | ☒ | The statistical test(s) used AND whether they are one- or two-sided<br>*Only common tests should be described solely by name; describe more complex techniques in the Methods section.* |
| ☒ | ☐ | A description of all covariates tested |
| ☐ | ☒ | A description of any assumptions or corrections, such as tests of normality and adjustment for multiple comparisons |
| ☐ | ☒ | A full description of the statistical parameters including central tendency (e.g. means) or other basic estimates (e.g. regression coefficient) AND variation (e.g. standard deviation) or associated estimates of uncertainty (e.g. confidence intervals) |
| ☐ | ☒ | For null hypothesis testing, the test statistic (e.g. *F*, *t*, *r*) with confidence intervals, effect sizes, degrees of freedom and *P* value noted<br>*Give P values as exact values whenever suitable.* |
| ☒ | ☐ | For Bayesian analysis, information on the choice of priors and Markov chain Monte Carlo settings |
| ☒ | ☐ | For hierarchical and complex designs, identification of the appropriate level for tests and full reporting of outcomes |
| ☐ | ☒ | Estimates of effect sizes (e.g. Cohen's *d*, Pearson's *r*), indicating how they were calculated |

*Our web collection on statistics for biologists contains articles on many of the points above.*

## Software and code

Policy information about availability of computer code

| | |
|---|---|
| Data collection | No software was used for data collection |
| Data analysis | GenomeStudio v2.0, StrandScript, CellRanger v5.0.0, RCA2, demuxlet, DoubletFinder v2.0.3, Seurat v3.2.3., GSEApy v0.10.4, Mfuzz v2.48.0, and custom codes (https://github.com/prabhakarlab/SCAN_COVID19) |

For manuscripts utilizing custom algorithms or software that are central to the research but not yet described in published literature, software must be made available to editors and reviewers. We strongly encourage code deposition in a community repository (e.g. GitHub). See the Nature Portfolio guidelines for submitting code & software for further information.

## Data

Policy information about availability of data

All manuscripts must include a data availability statement. This statement should provide the following information, where applicable:

- Accession codes, unique identifiers, or web links for publicly available datasets
- A description of any restrictions on data availability
- For clinical datasets or third party data, please ensure that the statement adheres to our policy

Raw sequencing data generated in this study are available via the European Genome-Phenome Archive (EGA): Study - EGAS00001005545 (https://ega-archive.org/studies/EGAS00001005545); Dataset - EGAD00001007995 (https://ega-archive.org/datasets/EGAD00001007995). Processed data are available via the Zenodo (https://doi.org/10.5281/zenodo.5153528).

# Research involving human participants, their data, or biological material

Policy information about studies with human participants or human data. See also policy information about sex, gender (identity/presentation), and sexual orientation and race, ethnicity and racism.

| | |
|---|---|
| Reporting on sex and gender | Clinical samples are obtained under the PROTECT protocol approved by the Singapore National Healthcare Group Domain-Specific Review Board (2012/00917). All participants provided written informed consent for sample collection and subsequent analyses. Findings are not specific for any sex (male/female). We performed single cell analysis on PBMCs in batches of 16 samples each, taking care to minimize batch-to-batch differences in age, sex and ethnicity |
| Reporting on race, ethnicity, or other socially relevant groupings | Clinical samples are obtained under the PROTECT protocol approved by the Singapore National Healthcare Group Domain-Specific Review Board (2012/00917). All participants provided written informed consent for sample collection and subsequent analyses. Findings are not specific for any ethinicity. This study has PBMCs mainly from Chinese, Indian, Malay ethnicities as well as a few individuals of other ethnicities (Bangladesh etch, categorized as Others in this study). We performed single cell analysis on PBMCs in batches of 16 samples each, taking care to minimize batch-to-batch differences in age, sex and ethnicity. |
| Population characteristics | We performed scRNA-seq and immune repertoire profiling on 286 peripheral blood samples collected longitudinally from an age- and sex-matched cohort of 112 patients, and documented disease duration and severity at the time of sample collection. We had 43 female and 69 male participants in the study. The study samples came from individuals of Malay (12), Indian (10), Chinese (69) and other (21) ethnicities. 73/108 (108 participants from 112 chosen after cell quality analysis) participants (68%) were sampled at least once during Days 1-8 |
| Recruitment | Clinical samples are obtained under the PROTECT protocol approved by the Singapore National Healthcare Group Domain-Specific Review Board (2012/00917). All participants provided written informed consent for sample collection and subsequent analyses. |
| Ethics oversight | Clinical samples are obtained under the PROTECT protocol approved by the Singapore National Healthcare Group Domain-Specific Review Board (2012/00917). All participants provided written informed consent for sample collection and subsequent analyses. |

Note that full information on the approval of the study protocol must also be provided in the manuscript.

# Field-specific reporting

Please select the one below that is the best fit for your research. If you are not sure, read the appropriate sections before making your selection.

☒ Life sciences ☐ Behavioural & social sciences ☐ Ecological, evolutionary & environmental sciences

For a reference copy of the document with all sections, see nature.com/documents/nr-reporting-summary-flat.pdf

# Life sciences study design

All studies must disclose on these points even when the disclosure is negative.

| | |
|---|---|
| Sample size | Peripheral blood samples from 112 COVID-19 patients with varying disease severity (108 after QC; Figure 1A). These samples were part of a larger cohort study of COVID-19 conducted in Singapore. Patients with serial blood samples in this cohort were identified, and individuals with a severe or critical outcome were matched by age (+/-5 years) and sex (exact) to patients with asymptomatic or mild outcomes. The number of COVID-19 patient samples for this study were consistently higher than the other studies performed in the same period. We chose to obtain patient samples where serial blood samples from the same patient were available. No statistical method was used to predetermine sample size. The sample size selected was sufficient as we had at least two distinct time points per individual and PBMCs were isolated from blood for downstream analysis. |
| Data exclusions | Samples with less than 200 single cells post-QC were excluded in the study |
| Replication | Prognostic markers were identified by comparing 11 Progressors to 52 Non-Progressors, sampled from Days 1-8 symptom onset. We validated our results using external cohorts as detailed in the manuscript. In addition, for single cell experiments, they were performed in batches of 16 individuals and in replicates which were used for sequencing downstream. Wet lab experiments on gene silencing are confirmed to be replicated. |
| Randomization | Samples were batched into pools of 16 for scRNA seq in a randomized manner, to control for batch effects arising due to age, sex or disease severity. |
| Blinding | Blinding was not relevant since we had to know disease severity at each time point before analysis. |

# Reporting for specific materials, systems and methods

We require information from authors about some types of materials, experimental systems and methods used in many studies. Here, indicate whether each material, system or method listed is relevant to your study. If you are not sure if a list item applies to your research, read the appropriate section before selecting a response.

## Materials & experimental systems

| n/a | Involved in the study |
|---|---|
| [x] | [ ] Antibodies |
| [ ] | [x] Eukaryotic cell lines |
| [x] | [ ] Palaeontology and archaeology |
| [x] | [ ] Animals and other organisms |
| [x] | [ ] Clinical data |
| [x] | [ ] Dual use research of concern |
| [x] | [ ] Plants |

## Methods

| n/a | Involved in the study |
|---|---|
| [x] | [ ] ChIP-seq |
| [x] | [ ] Flow cytometry |
| [x] | [ ] MRI-based neuroimaging |

# Eukaryotic cell lines

Policy information about cell lines and Sex and Gender in Research

| | |
|---|---|
| Cell line source(s) | HEK293T cells expressing human ACE2 were recieved from BEI resources (HEK-Ace2, #NR-52511) |
| Authentication | Characterized by BEI resources. We did not characterize it internally. |
| Mycoplasma contamination | Cell line were tested for mycoplasma contamination |
| Commonly misidentified lines (See ICLAC register) | No commonly misidentified cell lines were used in the study |
